# Atomic Imaging of Ion-Triggered Flexibility and Local Electric Field Response in Zeolite Rings

**DOI:** 10.1021/jacs.5c22979

**Published:** 2026-05-27

**Authors:** Qiang Chen, Zhao-Bin Ding, Pengfei Cao, Minghui Chu, Yichun Cai, Zhuoxi Li, Zongyu Sun, Penghan Lu, Janghyun Jo, Junwen Chen, Jianqiang Liu, Jian Zhao, Hans-Georg Steinrück, Joachim Mayer, Jordi Arbiol, Tongwen Yu

**Affiliations:** † School of Chemical Engineering and Technology, Institute of Green Chemistry and Molecular Engineering, 26469 Sun Yat-sen University, Zhuhai 519082, P. R. China; ‡ Ernst Ruska-Centre for Microscopy and Spectroscopy with Electrons, 28334 Forschungszentrum Jülich GmbH, Jülich 52425, Germany; § Institute for a Sustainable Hydrogen Economy (IHE), Forschungszentrum Jülich GmbH, Jülich 52428, Germany; ∥ Research Institute of Petroleum Processing, 58297 Sinopec, Beijing 100083, P. R. China; ⊥ School of Chemistry and Chemical Engineering, 12436 Nanjing University of Science and Technology, Nanjing 210094, P. R. China; # RWTH Aachen University, Institute of Physical Chemistry, Landoltweg 2, 52074 Aachen, Germany; ¶ Central Facility for Electron Microscopy, RWTH Aachen University, Aachen 52064, Germany; ∇ Catalan Institute of Nanoscience and Nanotechnology − ICN2 (CSIC and BIST), Campus UAB, Bellaterra, Barcelona, Catalonia 08193, Spain; ○ ICREA, Pg. Lluís Companys 23, 08010 Barcelona, Catalonia, Spain

## Abstract

Zeolite flexibility triggered by ion-exchange tuning pore geometry impacts the responsive interactions with guest species in selective adsorption and industrial catalysis. However, direct observation of their local atomic structure deformation and subsequent fundamental flexibility correlation between ions and atomic microenvironment remains elusive. Here, we report atomic-resolution imaging of the microscopic ring flexibility in ion-exchanged MFI zeolites, revealing how ion-induced electric field alterations drive the framework distortions. Specifically, extra-framework Ba^2+^ ions confined within the 10-membered rings (10-MRs) trigger a 0.5 Å channel expansion, which exceeds the 0.2 Å expansion induced by Na^+^ ions while maintaining the framework’s macroscopic rigidity. Using differential phase contrast imaging, we visualize the local coupling of the internal electric field between confined ions and the MFI framework. The combination of our theoretical analysis, which is based on the structure and the charge distribution, demonstrates that the electric field generated by the Ba^2+^ results in an 8° deformation in the nearby T–O–T bond angles, thereby distorting the 10-MR. The ion-exchange-driven 10-MR flexibility was further experimentally verified by the adsorption and separation of CO_2_, as this molecular size matches the expanded rings. This work resolves a long-term unsettled issue on the ion-exchange effect of pore size deformation in zeolites, offering insights into electric field-driven framework dynamics and introducing the intrinsic behavior of ions confined in zeolites.

## Graphical abstract

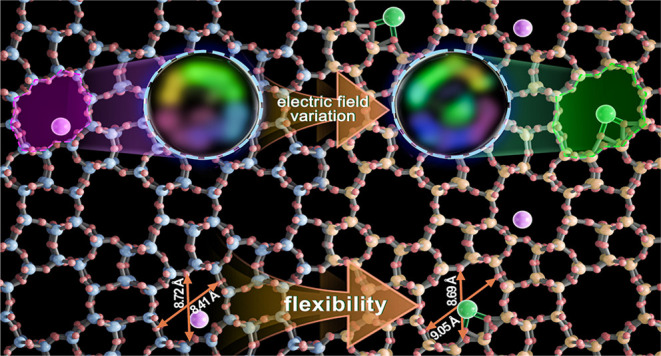

## 1 Introduction

Zeolites are crystalline aluminosilicates displaying periodic microporous architectures that can be fine-tuned for catalysis, adsorption, ion exchange, and membrane.^1–5^ Their intrinsic flexibility, including framework deformation and structure dynamics, renders minor pore adjustments (1–5 Å) by an external trigger such as changes in temperature or pressure, framework composition, and adsorption of molecules.^6–8^ Notably, ion exchange, which provides a versatile technology platform for fundamental investigations and industrial processes of extra-framework functional sites in diverse applications,^9,10^ has been experimentally proven to slightly regulate pore size, as observed in commercially available zeolites 3A, 4A, and 5A.^11,12^ Previous technologies for probing zeolite structural dynamics during ion exchange rely primarily on gas adsorption and X-ray diffraction (XRD). Gas adsorption provides an indirect, probe-dependent pore size measurement,^11^ while XRD yields averaged structural data lacking the spatial resolution to resolve specific local pore deformations induced by guest ions.^13,14^ Direct evidence of ion-exchange-triggered zeolite flexibility was somewhat unclear for more than 100 years^15^ because of the difficulty in characterizing local atomic structure in the electron beam-selective zeolites. Moreover, the charge compensation mechanism in zeolite ion exchange is strongly associated with the internal electric field, which bridges the gap between static crystallography and the material’s adaptive behavior. This relationship helps explain why cation-exchanged zeolites can exhibit different adsorption and reaction selectivities despite having identical framework topologies.^16,17^ However, the correlation between flexibility and internal electric field is rarely investigated. It is therefore crucial to develop direct atomic insights into the local flexibility of ion-exchanged zeolites.

State-of-the-art electron microscopy has emerged as the primary technology for the direct visualization of aperiodic local structures, including defects, interfaces, and deformations in zeolites at the atomic scale.^18–24^ Low-dose integrated differential phase contrast scanning transmission electron microscopy (iDPC-STEM) efficiently resolves the local zeolite structure, especially guest species confined in micropores.^25–29^ Pioneering works from Prof. Wei et al. on the iDPC-STEM characterization of host–guest interaction revealed benzene molecules diffusion in micropores exhibit zeolite subcell topological flexibility due to Si–O–Si angle distortion.^30^ Prof. Han et al. demonstrated that a four-dimensional STEM can precisely probe orientations of adsorbed *p*-xylene molecules within zeolites.^18^ Nevertheless, beyond the cyclic guest molecules, direct evaluation of the simplest guest ion-induced flexibility and the consequent atomic-scale trigger mechanism is not well clarified for zeolites. Here, we atomically visualize size variations of zeolite pores after the ion-exchange and systematically investigate the underlying trigger mechanism of ring flexibility by multiscale analyses. Direct-imaging iDPC-STEM reveals the size alteration of 10-MR, where Na^+^, Ba^2+^, and Cs^+^ ions are located in zeolites ZSM-5 (MFI type), which is also confirmed by XRD Rietveld refinement and density functional theory (DFT) calculation. Furthermore, the DPC-STEM images and X-ray absorption spectroscopy (XAS) results demonstrate that the enhanced internal local electric field due to extra-framework ions causes the O–T–O bond angle distortion within 10-MRs, thereby exhibiting the framework flexibility. We also conducted the adsorption and separation of CO_2_ to verify the 10-MR flexibility triggered by Na^+^ and Ba^2+^ ions.

## 2 Results and Discussion

Zeolite ZSM-5 possesses a three-dimensional channel system comprising an interconnected straight channel (5.3 Å × 5.6 Å) and a sinusoidal channel (5.1 Å × 5.5 Å) defined by 10-MRs.^31,32^ After ion exchange of Na-ZSM-5 with Ba^2+^ and Cs^+^ (Figures S1–S4), the Si/Al ratio (Table S1) and specific surface area (Table S2) of the resulting ZSM-5 remained nearly unchanged and preserved its typical nanosheet morphology (Figures S5 and S6). The atomic-scale iDPC-STEM image of Na-ZSM-5 (Figure 1A) directly observed Na^+^ existence in the straight channel, where T atoms were clearly imaged from the best imaging window of [010] projection (Figure S7). Four 10-MRs periodically appeared along the line with a total length of 5.01 nm in the intensity profile analysis, and they well matched with the standard crystallographic information on ZSM-5 (Figure 1B). A magnified image (Figure 1C) showed the presence of Na^+^ ions (indicated by red arrows) at the edge of the 10-MR wall, while the information transfer to approximately ∼1.7 Å (Figure 1D) enabled the observation of fine structural details at the atomic level (Figure S7). Given the sensitivity of iDPC to sample thickness and defocus (see simulation results in Figures S8–S10), a thin edge was selected for investigation. The experimental image displays a high degree of agreement with the simulation results, thereby confirming the reliability of the obtained results. After the Ba^2+^ ion-exchange process on Na-ZSM-5, the iDPC-STEM image (Figure 1E) of Ba-ZSM-5 distinctly showed the atomic-scale coexistence of Ba^2+^ and Na^+^ ions within straight channels, where the approximate locations of Ba^2+^ ion situated near the center. Comparison of the results with the simulated images (Figure S11A–D) shows good agreement in both contrast and spatial distribution. These results provide further evidence for the assignment that Ba^2+^ and Cs^+^ ions are effectively confined within the 10-MR channels following ion exchange. The profile analysis demonstrates the periodical appearance of the four 10-MR confined Ba^2+^ ions, whose total length (5.25 nm) is longer compared to that of Na-ZSM-5 along the same direction. It indicates a slight deformation of 10-MRs when the Ba^2+^ ion exchanges Na^+^ in ZSM-5, while the corresponding 10-MR structure continues to exhibit the standard crystallographic structure of ZSM-5 (Figure 1F). To better measure the varying geometries of 10-MRs, Length 1 is defined from the midpoint of Si_5_–Si_6_ to Si_10_–Si_1_, while Length 2 is defined from the midpoint of Si_2_–Si_3_ to Si_7_–Si_8_ (Figure S12). The size of Na^+^-occupied 10-MRs in Ba-ZSM-5 has been carefully measured as 8.7 and 8.4 Å for Lengths 1 and 2 (Figure 1G), respectively. In contrast, the values are 9.0 and 8.7 Å for Ba^2+^-occupied 10-MRs (Figure 1H). This size difference reveals that 10-MRs adapt to extra-framework ions, demonstrating the flexibility of 10-MRs^30^ during the ion-exchange process. The further statistical analysis in Figure 1I shows that the mean size of Na^+^-occupied 10-MRs was precisely measured to be 8.72 ± 0.01 Å and 8.41 ± 0.02 Å for Lengths 1 and 2, and compared to the size variation measured in Na^+^-occupied 10-MRs, the geometric size of Ba^2+^-occupied 10-MRs (9.05 Å × 8.69 Å) exhibits a more pronounced change. The mean values are 8.53 ± 0.01 Å and 8.61 ± 0.01 Å for unoccupied 10-MRs (Figure 1I). This size discrepancy indicates that 10-MRs adapt to extra-framework ions, while unoccupied ones shrink in one dimension to maintain the overall structure rigidity, thereby exhibiting 10-MR flexibility during the ion-exchange process. This observation suggests that zeolites, as a unique class of materials, possess local softness embedded within an overall rigid framework. Furthermore, these results reveal the distinct flexible deformation of the 10-MR pores during the ion-exchange process, providing the first direct evidence of such structural adaptations.

**Figure 1:**
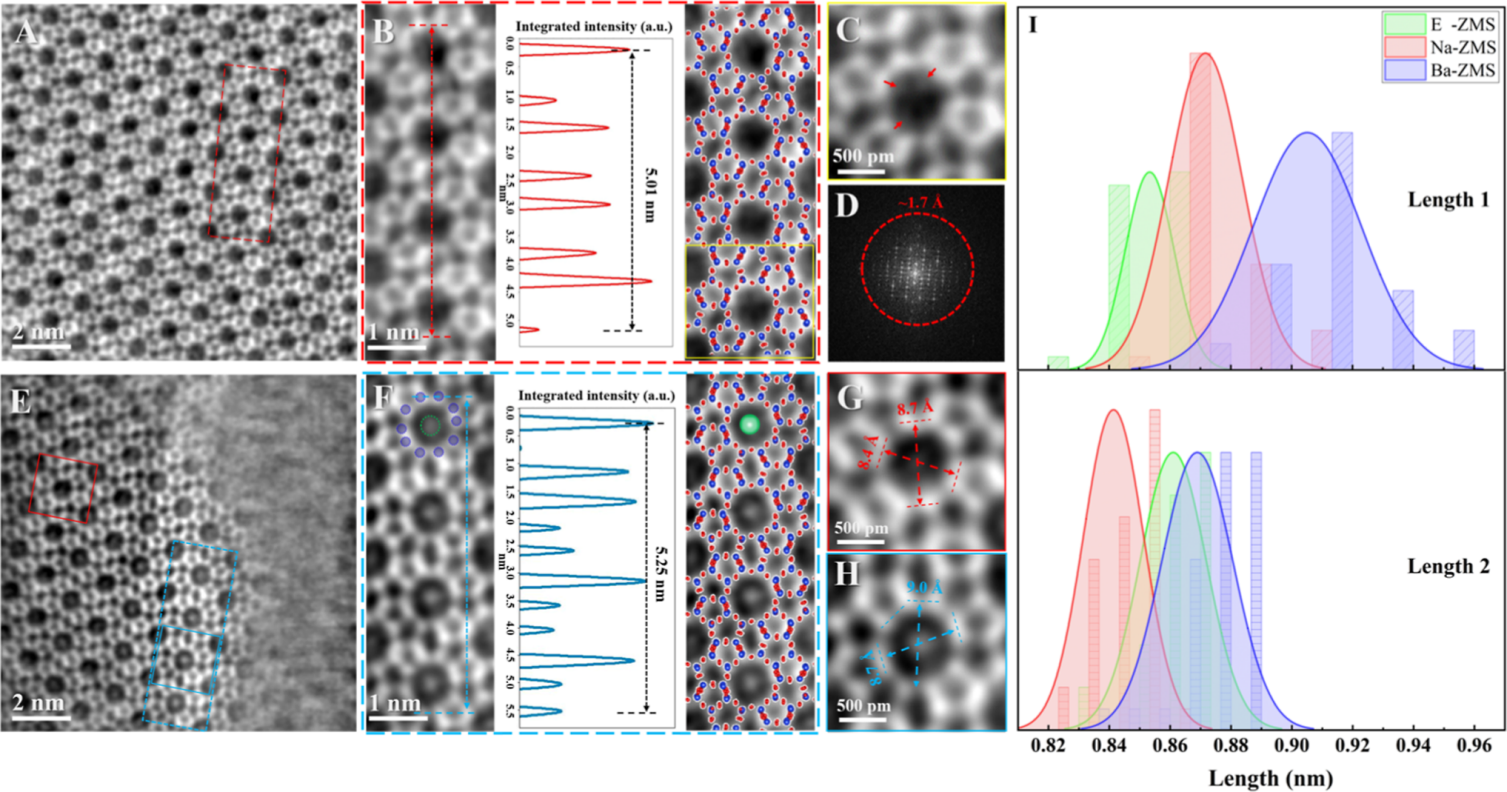
iDPC-STEM characterization of the ring flexibility of ion-exchanged zeolites. (A) Na-ZSM-5 image. (B) Left: image of the marked red area in (A), middle: the corresponding intensity profile of the defined line, right: the comparison between the left image and crystallographic information file (CIF) of ZSM-5. Red, blue, and green dots correspond to O, Si/Al, and Ba, respectively. (C) The magnified image of the marked area in the right yellow part of (B), and red arrows indicate the observed confined Na^+^ within the zeolite. (D) The corresponding FFT pattern of (C). (E) Ba-ZSM-5 image. (F) Left: image of the marked blue area in (E), middle: the corresponding intensity profile of the defined line, right: the comparison between the left image and the crystallographic information file (CIF) of ZSM-5. The magnified image of the marked red (G) and blue (H) areas in (E). (I) The statistical distribution of Length 1 and Length 2 for empty and Na^+^- and Ba^2+^-confined 10-MRs.

Next, a comparison between the observed patterns and the calculated data in Na/Ba-ZSM-5 (Figure 2A) using the XRD Rietveld refinement was conducted. This small *R*-factor (*R*_wp_, less than 10%) suggests successful Rietveld refinements, while lattice and atomic parameters confirm the unchanged symmetry (MFI framework with eight 10-MRs, *P*2_1_/*n*). Furthermore, parameters of the unit cell showed that the length is shortened by 0.003 Å along the *a* axis, but extended by 0.01 Å and 0.004 Å along the *b* and *c* axes, respectively, after ion exchange from Na-ZSM-5 to Ba-ZSM-5 (Figure 2A). Notably, the mismatch between the lattice parameters and pore size suggests flexibility of the ion-containing 10-MRs. DFT calculations showed 10-MR flexibility with distances of 8.50 Å and 9.33 Å in Na-ZSM-5, 8.54 Å and 9.38 Å in Ba-ZSM-5 (Figure 2B,C). These values confirm that the 10-MR distances in each individual unit cell are slightly elongated by the exchanged cations, consistent with the expansion revealed by iDPC imaging (see the Supporting Information for the discussion about the absolute values obtained from DFT and iDPC). These results further confirm the well-documented phenomenon of zeolite pore deformation^9,33^ when subjected to ion exchange at the unit cell scale. It is worth mentioning that iDPC-STEM characterization of zeolite flexibility at subunit cell resolution in response to ion exchange was the first to be highlighted.

**Figure 2:**
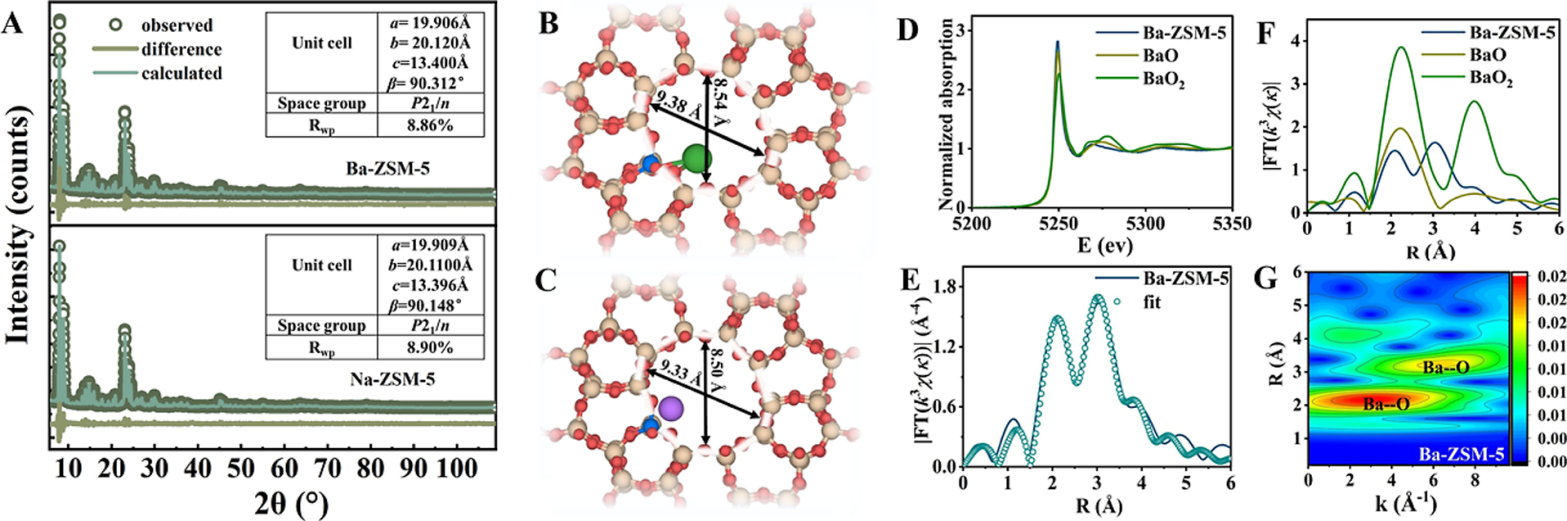
The X-ray characterization and DFT calculation of ring flexibility in ion-exchanged zeolites. (A) XRD Rietveld refinement of Na-ZSM-5 and Ba-ZSM-5; the comparison between the observed patterns and the calculated data. Insert: the corresponding lattice parameters. Note that the zero shift for Na-ZSM-5 and Ba-ZSM-5 is 0.002 and 0.012, respectively. (B, C) DFT calculation of the Ba^2+^- and Na^+^-10-MR size at top (B) and bottom (C), respectively. Red, light brown, blue, purple, and green dots correspond to O, Si, Al, Na, and Ba, respectively. (D to G) XAS characterization results of the Ba-ZSM-5 sample with BaO and BaO_2_ references: (D) XANES spectra at the Ba L_3_-edge; (E,F) Fourier-transformed *k* ^3^-weighted EXAFS spectra and the fitting result. (G) Wavelet transforms of Ba K-edge EXAFS contour plots for Ba-ZSM-5.

Subsequently, DFT calculations were further performed to investigate the structural distortion of the 10-MR, for which six distinct cation positions (Figures S13 and 14) were carefully selected. Taking one representative site as an example (Figure 2C), when Na^+^ is substituted by Ba^2+^, the distance between the cation (M^*n*+^) and the nearest T atom increases from 1.86 Å to 2.47 Å projected at the [010] plane (Figure S15). The phenomenon is also evident in the iDPC-STEM image (Figure S16). This trend corresponds well with the increasing ionic radii of the cations from 0.99 Å for Na^+^ to 1.35 Å for Ba^2+^.^34^ Furthermore, the difference Fourier maps for the approximate locations of Na^+^ and Ba^2+^ (Figure S17) show that the extra electron density representing Ba^2+^ appeared at a position slightly farther from the 10-MR than the Na^+^ electron density, suggesting that the possible location of the Ba^2+^ is somewhat more distant from the ring compared to Na^+^. The larger Ba^2+^ cations shift farther from the 10-MR ring edge and approach an oxygen atom on the adjacent side, facilitating the formation of a new Ba–O bond (Figures S13 and S14).

We then performed XAS to investigate the local structure and coordination microenvironment^35–38^ of Ba^2+^ in the straight channel. X-ray absorption near-edge structure (XANES) spectra (Figure 2D) show an increase in the white-line intensity in Ba-ZSM-5 compared to BaO and BaO_2_ references. The spectra resemble the BaO reference more closely. This unique coordination state can be attributed to the similar yet distinct geometry of the surrounding oxygen atoms in Ba-ZSM-5. Extended X-ray absorption fine structure (EXAFS) studies (Figure 2F) exhibit a prominent peak at 2.09 Å that is contributed by Ba–O scattering paths. This further demonstrated that the Ba species is coordinated by framework oxygen atoms. The EXAFS fitting results and corresponding parameters (Table S3) showed that the average Ba–O distance is 2.78 Å, which is close to the distance in BaO (2.77 Å). This suggests the formation of the Ba–O bond with the framework oxygen atoms in 10-MRs, which is consistent with the DFT results. Moreover, the coordination number of the Ba–O bond is 2, and two other neighboring oxygen atoms exist at a distance of 3.41 Å. Similar results were confirmed in the wavelet transform analyses of the Ba K-edge data (Figures 2E,G and S18).

To further prove the ion-induced ring flexibility, the iDPC-STEM imaging of larger Cs^+^ ion exchange of ZSM-5 (Figure 3A,B) was performed. We observed that the pore size of a Cs^+^-contained straight channel in the magnified image is 9.1× 8.4 Å (Figure 3C), indicating that length 1 is extended by 0.4 Å, while length 2 remains nearly unchanged compared to Na-ZSM-5. Moreover, it extended by +0.1 and −0.3 Å compared to Ba-ZSM-5, indicating the ionic size (Cs^+^ ionic radius of 1.67 Å is bigger than that of Ba^2+^, which is 1.35 Å) has no major effect on the 10-MR flexibility. Additionally, the XRD Rietveld refinement and the extracted unit cell parameters (Figure 3D) show that the lattice lengths vary by −0.002, −0.011, and +0.002 Å along the *a*, *b,* and *c* axes, respectively, relative to Na-ZSM-5. Furthermore, the DFT analysis (Figure S19) also reveals a structural distortion with 8.58 Å × 9.34 Å in Cs-ZSM-5 (Figure 3E), where the distance between the Cs^+^ and the nearest T atom increases to 3.07 Å (Figure S20), compared to that in Na-ZSM-5 with the distance of 1.86 Å (Figure S15). It is consistent with the difference Fourier maps analysis (Figure S21) that shows the extra electron density of Cs^+^ appears at a position farther from the 10-MR, indicating that Cs^+^ is farther from the ring compared to Na^+^. These results reveal that local ring flexibility and overarching framework rigidity allow for the accommodation of different ions such as Na^+^, Ba^2+^, and Cs^+^ with different radii, suggesting that the larger size of the cation does not always result in an even enlargement of the zeolite pore.

**Figure 3:**
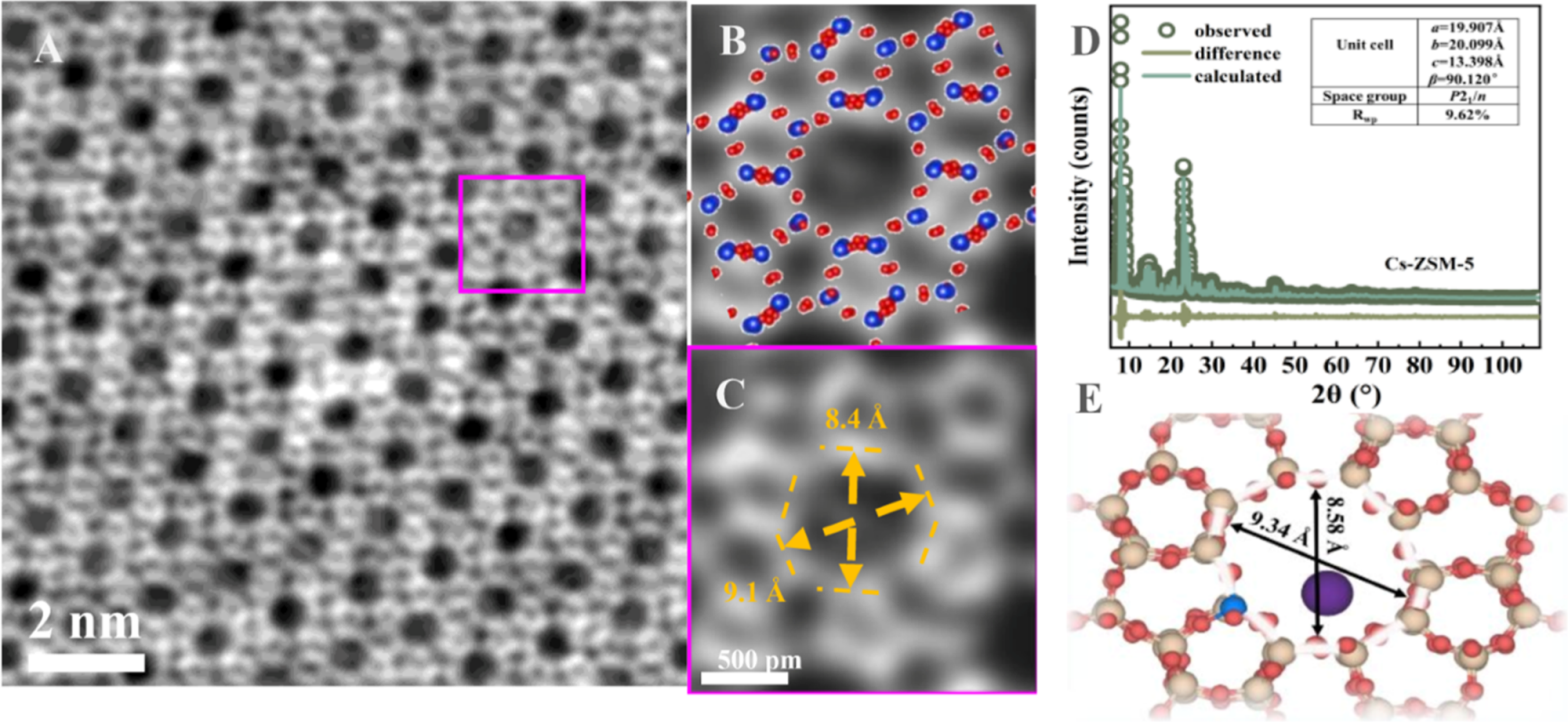
The iDPC-STEM, XRD Rietveld refinement, and DFT calculation of ring flexibility in Cs^+^ion-exchanged zeolites. (A) iDPC-STEM characterization of the ring flexibility of Cs-ZSM-5. (B) Image of the marked pink area in (A), and the comparison between the image and ZSM-5 CIF. Red and blue dots correspond to O and Si/Al, respectively. (C) The Cs^+^-containing 10-MR size measured. (D) The XRD Rietveld refinement of Cs-ZSM-5. Note that the zero shift for Cs-ZSM-5 is 0.008. (E) DFT calculation of the Cs^+^-10-MR size. Red, light brown, blue, and dark purple dots correspond to O, Si, Al, and Cs, respectively.

Direct observation of ring deformation indicated interactions between guest ions and the zeolite framework. Compared to Na-ZSM-5, the introduction of exchanged ions such as Ba^2+^ and Cs^+^ disturbs the intrinsic local electric field of the framework due to their distinct charge characteristics, which should greatly impact zeolite behavior. Here, we employed the DPC STEM technique to directly visualize the atomic electric fields of Na-, Ba-, and Cs-ZSM-5 along the [010] direction (Figures 4A–D and S22–S23). Furthermore, we applied theoretical electrostatic analysis based on structures calculated by DFT to deepen our microscopic understanding. The projected electric field magnitude and color map provide insights into the relative strength of the electric field at each location in the corresponding DPC image. In theory, an isolated atom exhibits a radially outward electric field with a perfect circle-shaped disc, but this symmetry can be disrupted by the electric field of an adjacent atom due to partial cancellation. Thus, the in-plane electric field mapping of ZMS-5 resolves the superposition of electric fields from all neighboring atoms. Notably, the net electric field strength in empty rings, such as 5-MRs, 6-MRs, and 10-MRs without ions (Figure 4C, highlighted in a yellow circle region), exhibits an inward radial electric field within the structure, approaching a net zero strength at the high symmetry points of the structure with a dark contrast. This finding is consistent with the DPC simulation results (Figure S10I) and the extremely low electric field at the void center of a bare pore projected on the [010] plane, which is theoretically predicted to be 12 V/nm. However, the introduction of extra-framework ions in 10-MRs generates new internal electric fields coupled with each other, leading to a charge density redistribution within the local 10-MR framework. Figure S22 shows a representative coupling electric fields map in the interstitial region between Na^+^ and adjacent atoms in 10-MRs. The coupling electric field appears close to the 10-MR framework, confirming that the position of Na^+^ is near the framework. In contrast, sharper electric fields of Ba^2+^ and Cs^+^ ions are visible inside the 10-MRs as the distance between the ions and the framework increases (Figures 4B and S23), consistent with the DFT results. In addition, we have performed DPC simulations for ZSM-5, Na-ZSM-5, Ba-ZSM-5, and Cs-ZSM-5 (Figure S11F–H). The results show that the presence of Na^+^, Ba^2+^, and Cs^+^ leads to distinct local electric-field distributions within the MFI framework and forms the local coupling internal electric field between confined ions and the MFI framework. In particular, the ion-induced electric-field strength follows the trend Ba^2+^ > Cs^+^ > Na^+^, which is consistent with both our experimental DPC observations and DFT calculations. Unlike the radially symmetric electric field of isolated atoms, the electric field distribution near the Ba^2+^ ion is asymmetrically distorted because of partial cancellation by the outward electric field of the framework along the directions with shorter distances to adjacent oxygen atoms (enlarged map in Figure 4D). For instance, the electric field at the midpoint of the M–O6 bond is 43% stronger than that at its symmetrically inverse position (124 vs 86 V/nm) projected on the [010] plane (Figure S24). Furthermore, the strength of the coupling electric field is correlated with the distance between Ba^2+^ and the framework. When the relative distance is too short, the coupling electric field approaches to nearly zero in a certain direction (e.g., direction 1 in Figure 4D). Conversely, if the relative distance is too large, the electric field of the Ba^2+^ and the framework becomes independent, forming an uncoupled gap region between them (direction 3 in Figure 4D). For example, theoretical electrostatic analysis reveals that the strength of the electric field induced solely by Ba^2+^ at the adjacent O site is approximately 24 V/nm, while that at the oxygen atom located on the opposite point of the 10-MR is less than 5 V/nm (Figure S25). Therefore, it has little effect on the bond angles far away from the cation. In a specific direction, the electric field of Ba^2+^ is only partially canceled due to the framework. A continuous coupling electric field is formed from Ba^2+^ ions toward the framework oxygen atoms (direction 2 in Figure 4D). The consequences of these electric fields are then investigated by analyzing six distinct structures for Na-ZSM-5, Ba-ZSM-5, and Cs-ZSM-5, respectively (Table S4). According to the bond length and bond angle values provided by DFT calculations, despite the substitution causing only minor changes in Al–O and Si–O bond lengths (<0.05 Å; Figure S26), O–T–O and T–O–T angles near the cations shift by up to 15° (Figure 4E,F), while most others vary by less than 5° (Figures S27 and S28). We picked up one of the six structures for ZSM-5, Na-ZSM-5, and Ba-ZSM-5 for detailed analysis to investigate the local bond angle change pattern. This analysis shows that introducing Na^+^ and Ba^2+^ reduces the two O-T-O bonds by 2° and 6.3° for Na-ZSM-5 (Figure S29), and by 7.6° and 6.0° for Ba-ZSM-5 (Figure 4G). In addition, the Ba^2+^ has a generally more pronounced effect on the bond changes than Na^+^ and Cs^+^, also indicating that the ionic size is not the main role for the ring flexibility. The two T-*O*-T bonds also decreased by up to 8.2° with the introduction of the cation (Figure 4H). These results reveal that changes in the electrostatic force on the nearby O and T atoms distort specific T-*O*-T and O-T-O angles, thereby expanding nearby rings depending on the charge and position. Therefore, the electric field generated by the cation is the primary cause of the flexibility. The Bader charge analysis of the electric field distribution at specific points on the 10-MR ring is listed in Figure S30. Furthermore, the electron localization function results (Figure S31) revealed changes in the local electric field upon Ba^2+^ substitution, indicating partial covalent character in the Ba–O interaction. These results demonstrate that the presence of cations introduces a dynamic interplay between framework softness and electrostatic constraints, indicating the zeolite flexibility as a tunable property that can be engineered through cation type, density, and location.

**Figure 4:**
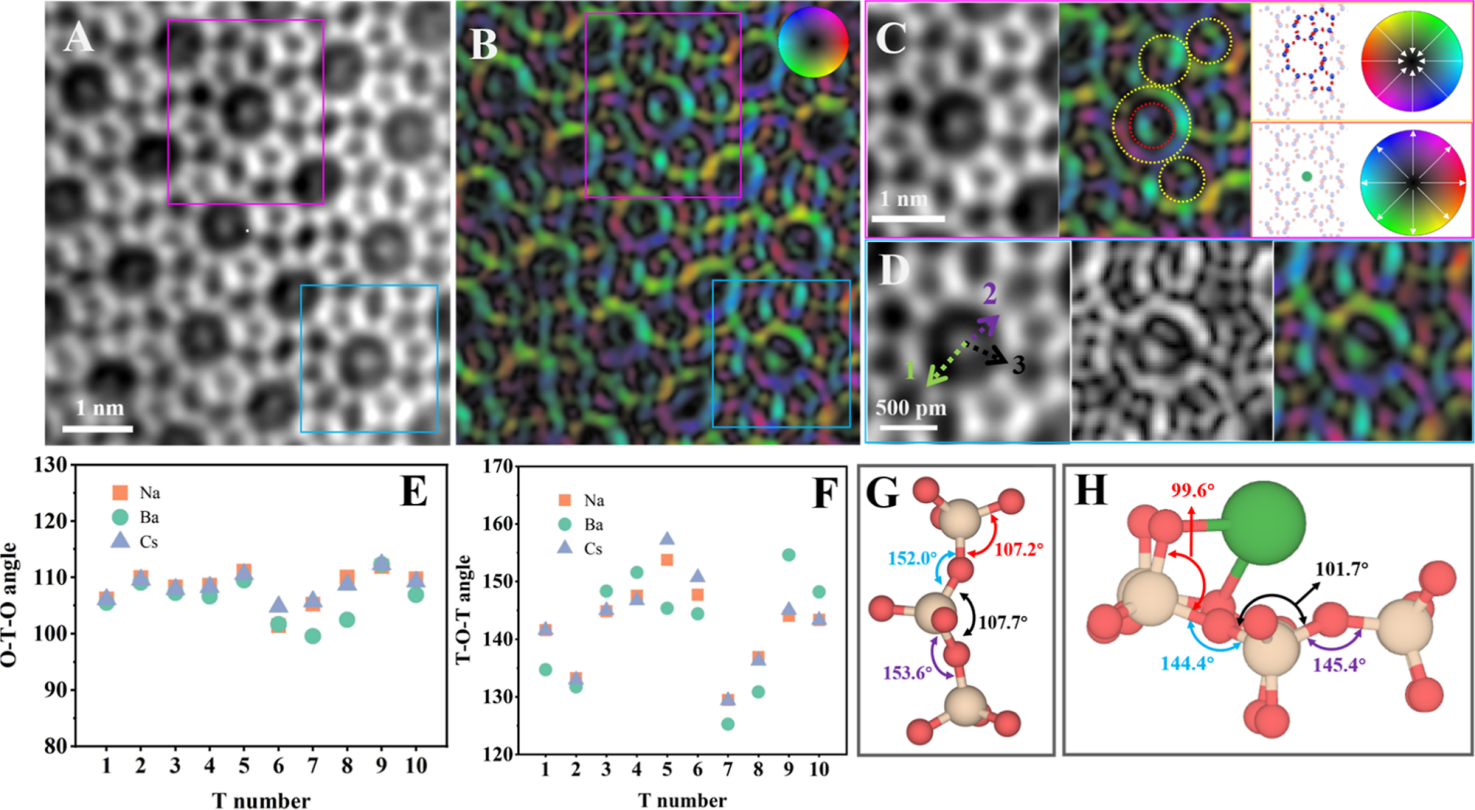
Investigation of the electric field trigger mechanism for ring flexibility via DPC-STEM and DFT calculation. (A–D) iDPC-STEM image (A) and the corresponding DPC map (B) of Ba-ZSM-5, the iDPC-STEM image, and the corresponding DPC maps (C,D) of the specific 10-MRs containing the Ba^2+^ ion. (E,F) Distributions of O-T-O angle (E) and T-*O*-T angle (F) between adjacent tetrahedral TO_4_ within 10-MR with different ions located at the 8th position in DFT calculation. (G,H) The bond angles in the detailed 10-MR pore structure of empty ZSM-5 (G) and Ba-ZSM-5 (H). Red, light brown, and green dots correspond to O, Si, and Ba, respectively.

As a proof of concept for the ring flexibility of ion-exchanged zeolite, the effect of CO_2_ capacity on Ba^2+^ concentration in ZSM-5 was measured by the adsorption isotherms. The efficient size of Ba^2+^-occupied 10-MR is 0.28 nm when rigid, but expands to 0.33 nm due to ring flexibility observed by atomic imaging, which is the same as the kinetic diameter of CO_2_ (Table S5). Moreover, only the 10-MR channels can accommodate CO_2_, whereas smaller 6-MRs (0.26–0.28 nm) and 5-MRs (0.18–0.20 nm) apertures cannot. Presumably, in a rigid 10-MR framework, the introduction of high concentrations of Ba^2+^ reduces the available pore space. In such a scenario, the 10-MR channels behave as if they are partially blocked, which in turn leads to a decrease in the CO_2_ adsorption capacity of the zeolite. Otherwise, it implies a flexible 10-MR. As shown in Figure S32, CO_2_ adsorption isotherms of Ba-ZSM-5 at 298 K exhibit that CO_2_ uptake remains consistent at ∼40 cm^3^/g, despite high Ba^2+^ concentrations measured by X-ray fluorescence spectroscopy (XRF). It reveals that the Ba^2+^ occlusion in most of the 10-MRs has minimal impact on CO_2_ adsorption capacity, underscoring 10-MRs’ flexibility for the accommodation of both Ba^2+^ and CO_2_. In addition, the breakthrough experiments for CO_2_/N_2_ were chosen because N_2_ has minor interaction with zeolite micropores. Figures S33 and S34 show similar separation trends of CO_2_/N_2_ in Na-ZSM-5 and Ba-ZSM-5, but the latter displayed a longer penetration interval time. This may be because CO_2_ exhibits a stronger affinity for Ba^2+^ than for Na^+^. The adsorption rate measurements are consistent with this observation (Figure S35), showing slightly faster CO_2_ uptake on Ba-ZSM-5 than on Na-ZSM-5. The adsorption of CO_2_ is predominated by physisorption through M^n+^···O=C=O interactions that rely on electrostatic interactions with quadrupole CO_2_.^39^ It implies that direct electrostatic interactions can immobilize cations into the pore, altering the effective size of the pore window. Moreover, the electric field significantly affects the attraction of CO_2_ when passing through the cations, which finally determines the efficiency of separation. For this purpose, we theoretically calculated the electric field intensity at the center of the pore that contains different types of cations. The total electric fields projected on the [010] direction of Na-ZSM-5 and Ba-ZSM-5 are 12 and 22 V/nm, respectively. In particular, the fields generated by the cations are 8 and 19 V/nm (Figure S36). It reveals that the dominant difference of the intensity is the field generated by cations. The Ba^2+^ provides more than twice the electric field of Na^+^, which agrees with the doubled charges on Ba^2+^, and Ba^2+^ is 0.61 A closer to the center of the pore than Na^+^. Therefore, under the presence of CO_2_ and Ba^2+^, a stronger electric field intensity leads to enhanced flexibility, thereby allowing Ba^2+^-containing 10-MRs to facilitate the passage of CO_2_.

## 3 Conclusions

In summary, we present the imaging of ring flexibility in zeolites induced by ion exchange and elucidate the atomic-scale flexible responses to internal electric fields. The ion-exchanged Na^+^, Ba^2+^, and Cs^+^ were atomically imaged within MFI 10-MRs, revealing the geometric variations of opening pores for ion accommodation. Notably, Ba^2+^ ions induce a 0.3 Å greater expansion of 10-MRs compared to Na^+^ ions, while maintaining overall framework rigidity. Our findings reveal how extra-framework ions generate localized electric fields that distort O–T–O bond angles of 10-MRs. This establishes a mechanistic link between ion-specific framework flexibility and electric field redistribution, which was further confirmed by the adsorption and separation of CO_2_, which extend the host–guest interaction to the coupled framework–cation–guest dynamics. This work not only provides the direct evidence of ion-exchange influence on the exact pore size, but also reveals the ring flexibility correlation between ion location or size, local electric fields, and bond-angle distortions. These insights could help in enhancing the understanding of ion-selective ring topological flexibility in microporous materials from the standpoint of electric field redistribution, establishing design principles for next-generation breathing zeolites for gas separation, gated diffusion, smart molecular recognition, and shape-selective zeolite catalysis.

## Supplementary Material



## Notes

Full experimental materials, methods, characterizations, computational details, and additional figures, tables, and references as mentioned in the text (PDF)
