# Supplementary material for: Atomic Imaging of Ion-Triggered Flexibility and Local Electric Field Response in Zeolite Rings
Source: J Am Chem Soc. 2026 May 27;148(22):22504–11. doi: 10.1021/jacs.5c22979 (PMC13266982; doi:10.1021/jacs.5c22979)
Supplement: Supplementary file 1 [file ja5c22979_si_001.pdf]

Supplementary Materials for

Atomic Imaging of Ion-Triggered Flexibility and Local Electric Field Response in Zeolite Rings

Qiang Chen[1]†*, Zhaobin Ding[1]†, Pengfei Cao[2,3]*, Minghui Chu[1], Yichun Cai[1], Zhuoxi Li[1], Zongyu Sun[1], Penghan Lu[2], Janghyun Jo[2], Junwen Chen[4], Jianqiang Liu[4], Jian Zhao[5], Hans-Georg Steinrück[3,6], Joachim Mayer[2,7], Jordi Arbiol[8,9]*, Tongwen Yu[1]*

[1]School of Chemical Engineering and Technology, Institute of Green Chemistry and Molecular Engineering, Sun Yat-sen University, Zhuhai 519082, P. R. China.

[2]Ernst Ruska-Centre for Microscopy and Spectroscopy with Electrons, Forschungszentrum Jülich GmbH, Jülich 52425, Germany.

[3]Institute for a sustainable Hydrogen Economy (IHE), Forschungszentrum Jülich GmbH, Jülich 52428, Germany.

[4] Research Institute of Petroleum Processing, Sinopec, Beijing, 100083, P. R. China.

[5] School of Chemistry and Chemical Engineering, Nanjing University of Science and Technology, Nanjing 210094, P. R. China.

[6] RWTH Aachen University, Institute of Physical Chemistry, Landoltweg 2, 52074 Aachen, Germany

[7] Central Facility for Electron Microscopy, RWTH Aachen University, Aachen 52064, Germany.

[8] Catalan Institute of Nanoscience and Nanotechnology (ICN2), CSIC and BIST, Campus UAB, Bellaterra, Barcelona, Catalonia 08193, Spain.

[9] ICREA, Pg. Lluís Companys 23, 08010 Barcelona, Catalonia, Spain

†These authors contributed equally to this work.

*Corresponding author. Email: chenqiang3@mail.sysu.edu.cn (Q.C.); p.cao@fz-juelich.de (P.C.); arbiol@icrea.cat (J.A.); yutw@mail.sysu.edu.cn (T.Y.)

The PDF file includes:

Materials and Methods

Figures. S1 to S36

Table S1 to S5

References 1 to 12

Materials and Methods

Sodium hydroxide (NaOH, >98%, Macklin), Tetrapropylammonium hydroxide (TPAOH, 40wt%, Tci), isopropanol (IPA, >99.5%, Aladdin), tetraethyl orthosilicate (TEOS, 99.99%, Aladdin), urea (BC Grade, Sangon Biotech), Aluminum nitrate nonahydrate ($Al(NO_3)_3·9H_2O$, AR, 99.0%, Macklin), Cesium choride (CsCl, 99.99%, Aladdin), barium chloride dihydrate ($BaCl_2·2H_2O$, 99.99%, Aladdin). All chemicals are used as received.

Synthesis of Na-ZSM-5

Na-ZSM-5 was synthesized via a hydrothermal method. Initially, tetrapropylammonium hydroxide (TPAOH, 40% in water, 8.19 g), iso-propanol (IPA, 0.1 g), NaOH (0.1g), urea (2.0 g), and $Al(NO_3)_3 \bullet 9H_2O$ (0.3g) were sequentially added to 18.4 g of deionized water at room temperature to form a mixed solution. Subsequently, TEOS was dropwise added into the solution to ensure thorough dispersion, followed by magnetic stirring at 600 rpm for 8 h. The resulting mixture was transferred to an autoclave equipped with a polytetrafluoroethylene (PTFE) lining and heated to 180 °C at a rate of 15 °C/min, and then maintained for crystallization at 180 °C for 48 h. After crystallization, the sample was rapidly cooled to room temperature by cold water and washed via centrifugation with deionized water until neutral. The sample was then transferred to a muffle furnace, where it was calcined to 550 °C at a rate of 2 °C/min and maintained for 5 h to remove the templates, ultimately obtaining required Na-ZSM-5.

Synthesis of Ba-ZSM-5 and Cs-ZSM-5

For the synthesis of ion exchanged Ba-ZSM-5, 4.88g barium chloride dihydrate ($BaCl_2·2H_2O$) was added to 20 ml of deionized water and fully dissolved to form 1mol/L of barium chloride solution. Then 1 g of synthesized Na-ZSM-5 was added to above solution under stirring at 60 °C for 2 h. To ensure a sufficient cation exchange process being performed, the mixture was then centrifuged and followed by adding the

new $BaCl_2$ solution to continue to stir for 2 h at 60 °C. Such ion exchange procedure was repeated for 3 times. The obtained mixture was filtered, then washed with deionized water several times, followed by dried at 100 °C overnight. The resulting product was transferred to a muffle furnace, where it underwent calcination at 550 °C for 5 h to afford Ba-ZSM-5. Ba-ZSM-5 (0.1%) and Ba-ZSM-5 (0.5%) were synthesized with the same methods, except that the concentrations of barium chloride solution were changed from 1 mol/L to 0.1 mol/L and 0.5 mol/L. The ion exchange processes of CsCl to obtain Cs-ZSM-5 was similar to the above with the 1mol/L of CsCl solution.

Experimental Characterization Details:

The characterization of X-ray powder diffraction was performed on the Rigaku Ultima IV instrument. The Brunauer-Emmett-Teller (BET) tests were conducted on a BET analyzer (micromeritics ASAP 2460). Scanning electron microscopy (SEM) was taken on an instrument of the field-emission FEI Quanta FEG 250 microscope with the operation at an acceleration voltage of 20 kV. The ratios of Si/Al in three different ZSM-5 zeolites were determined by the instrument of Philips Magix-601 X-ray fluorescence (XRF) spectrometer. XRD patterns characterizations for Rietveld refinements were collected under room temperature by the using a Bruker D8 ADVANCE diffractometer (Cu K_{α} radiation, step scanning mode， 40kV, 40mA). Rietveld refinements were used to analyze the XRD data with the TOPAS software. The breakthrough experiment was carried out on the BSD-MAB at 298 K (water bath) and 1 atm.

TEM characterization:

The iDPC-STEM experiments were conducted using an FEI Titan G2 80-200 microscope equipped with a Cs-probe corrector and a HAADF STEM detector. The microscope was operated at 200 kV and the beam convergence was set to 17.6 mrad to acquire iDPC and HAADF images simultaneously. The images were collected by a four-quadrant DF4 detector after probe aberration corrected using a standard cross-grating specimen. The collection angles were 9-34 mrad for iDPC-STEM and 37-200

mrad for HAADF STEM. The iDPC-STEM image simulation was performed by using Dr. Probe software[1]. Multislice simulations encompass the relationship between image quality and various thicknesses, defocus, and resolutions. In consideration of the sensitivity of iDPC to sample thickness and defocus, a thin edge was selected for investigation. And calcination during sample preparation and plasma-treatment prior to iDPC-STEM were conducted to clean the surface of studied zeolites to avoid the surface contamination. Elemental maps were taken by energy-dispersive X-ray spectroscopy (EDX) using four large-solid-angle symmetrical Si drift detectors. Si, Al, O and Na K-lines, along with the Ba and Cs L-lines were used to analysis the elemental distributions and compositions of the samples. The quantification error is ±2 at.%. Electron energy loss spectrum (EELS) was performed to determine the relative sample thickness using a post-column energy filter system (Enfinium ER 977, Gatan Inc., Pleasanton, CA, USA).

To determine relative sample thickness, we use the log-ratio (relative) method[2]. Following Poisson statistics, the ratio of zero-loss electrons to the total transmitted intensity gives a relative measure of the specimen thickness in units of the local inelastic mean free path λ.

$$t/\lambda = -\ln(I_o/I_t)$$

Where:

t = specimen thickness

λ = inelastic mean free path (IMFP)

I_0= integrated intensity of zero-loss peak

I_t = integrated intensity of entire EELS spectrum

t/λ is the mean number of scattering events per incident electron.

In this research, this was computed easily from the low-loss spectrum via DigitalMicrograph software (Gatan, Inc.). Specifically, clicking the “Thickness” button located in the EELS processing palette in the “Techniques” panel, and t/λ will be obtained for the line profile region.

The IMFP can be calculated by using semi-empirical form from Ray Egerton:

$$\lambda = \frac{106E_0}{\rho Z \ln\left(\frac{2\beta E_0}{E_p}\right)}$$

Where:

E_0 = beam energy (keV)

E_p = plasmon energy (~23 eV for silicates)

β = collection semi-angle (radians)

ρ = density

Z = effective atomic number

The breakthrough experiments:

The length of the column was 67.3mm, the rate of feed gas was 2 mL/min, consisting of 1.7 mL/min N_2 and 0.3 mL/min CO_2, and packed zeolite particles was powder state. glass column with 1mm inner diameter was used to enforce this breakthrough test.

DFT calculations:

Periodic density functional theory (DFT) calculations were conducted for ZSM-5, Na-ZSM-5, Ba-ZSM-5, and Cs-ZSM-5 structures (denoted as M-ZSM-5 in the following description) with density functional theories. The Perdew–Burke–Ernzerhof (PBE) exchange-correlation functional[3], combined with Grimme's D2 dispersion corrections[4], was employed to accurately capture van der Waals (vdW) interactions. This combination has been validated by Maestri and Iglesia[5] to reliably model vdW interactions between adsorbates and siliceous zeolite frameworks. The periodic wavefunctions were expanded using a plane-wave basis set with a kinetic energy cutoff of 816 eV, and ultrasoft pseudopotentials were used to describe core electrons. Due to the large size of the simulation cell (288 atoms for siliceous ZSM-5), Brillouin zone sampling was limited to the Γ-point. The electronic energy convergence threshold was

set to 1.36×10^{-7} eV (1.0×10^{-8} Ry). All DFT calculations were performed using the Quantum ESPRESSO software package[6].

M-ZSM-5 models were constructed by substituting two randomly selected Si atoms, located in different straight channels, with Al atoms, resulting in a Si:Al ratio of 42. Eight distinct cation positions were evaluated. In Na-ZSM-5 and Cs-ZSM-5 systems, the framework's net negative charge, resulting from Al substitution, was compensated by placing an additional Na^+ in the opposite channel. Structural optimizations were performed by relaxing both atomic positions and cell parameters until the residual forces on all atoms were below 0.02 eV·$Å^{-1}$.

To analyze the localization of electrons around the cations—indicative of M–O bond character—the localized orbital locator (LOL) function was computed[7]. Bader charge analysis[8] was used to assess the valency of atoms within the 10-membered ring (10-MR). Charge density differences were evaluated using the expression:

$$\Delta\rho = \rho(\mathrm{M}^{\mathrm{n}+} - \mathrm{ZSM5}^{-}) - \rho(M^{n+}) - \rho(\mathrm{ZSM5}^{-})$$

where ρ denotes the electron density, M^{n+} represents the cation, and $ZSM5^-$ represents the surrounding framework atoms. Framework structures were extracted from the periodic models and terminated with hydrogen atoms for cluster-level analysis. These charge density calculations were performed with the PBE functional and def2-TZVP basis set using the ORCA software package[9].

Atomic measurement techniques comparison:

This work combines density functional theory (DFT), low-dose integrated differential phase contrast scanning transmission electron microscopy (iDPC-STEM), and Rietveld refinement to determine the atomic structure of exchanged zeolites. These three techniques probe structural information at different length scales and under different averaging conditions, which inevitably leads to differences in the derived absolute structural parameters.

DFT: Periodic DFT calculations explicitly solve the Schrödinger equation and provide accurate geometric and electronic structures for a ZSM-5 unit cell containing 288 atoms. As a result, DFT yields atomically resolved local structural variations within a single unit cell after cation introduction. The calculated pore size and shape therefore represent a specific local configuration and are not averaged over other pores or unit cells in the experimental sample, where cations may occupy different sites.

iDPC-STEM: iDPC-STEM provides a local projected image of a straight channel, with contrast contributions from all 10-membered ring (10-MR) edges along the [010] crystallographic direction. The signals arise from electron scattering by T–O bonds and other ions in the projected column.

Rietveld refinement: Rietveld refinement yields atomic positions averaged over a macroscopic sample volume (typically on the order of millimeters, e.g., 6 × 24 × 0.04 mm). Consequently, it cannot capture local structural heterogeneity unless the sample is a nearly perfect single crystal.

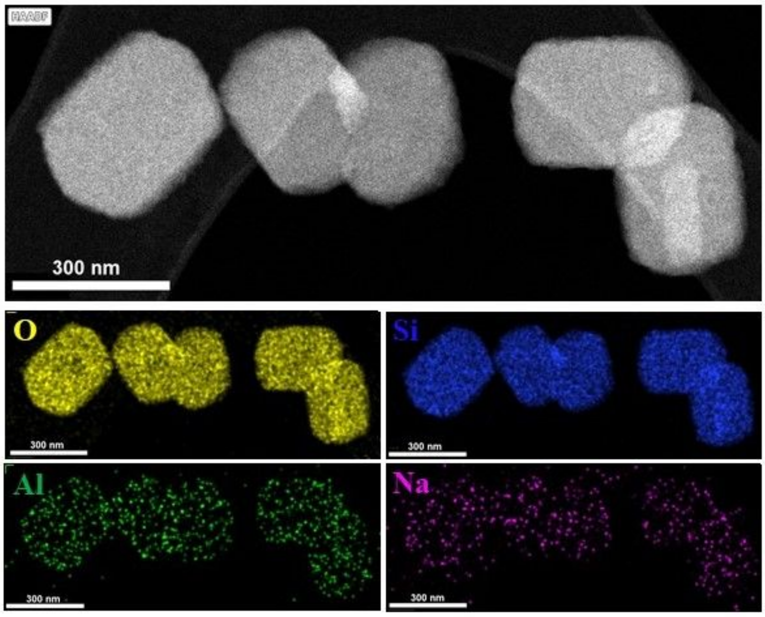

Figure S1: HAADF STEM and EDX-mapping of Na-ZMS-5

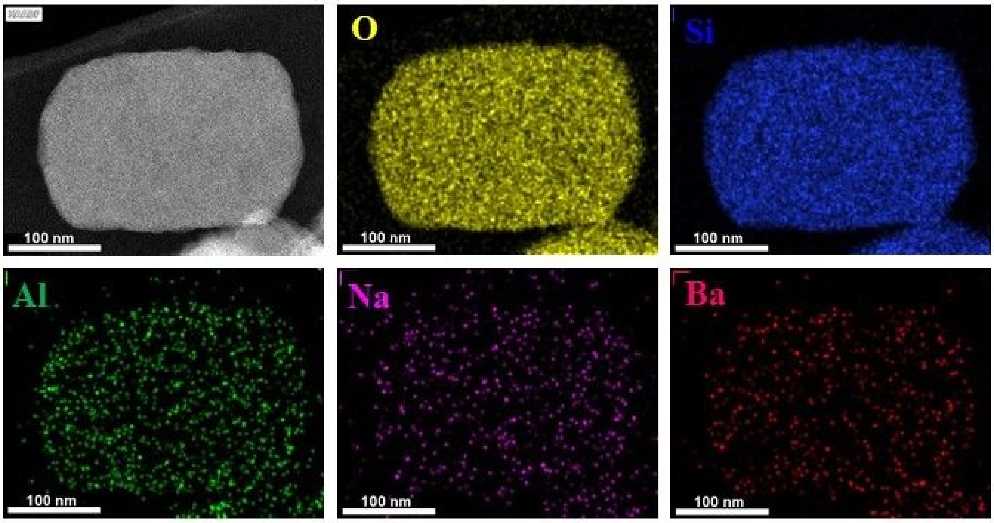

Figure S2: HAADF STEM and EDX-mapping of Ba-ZMS-5

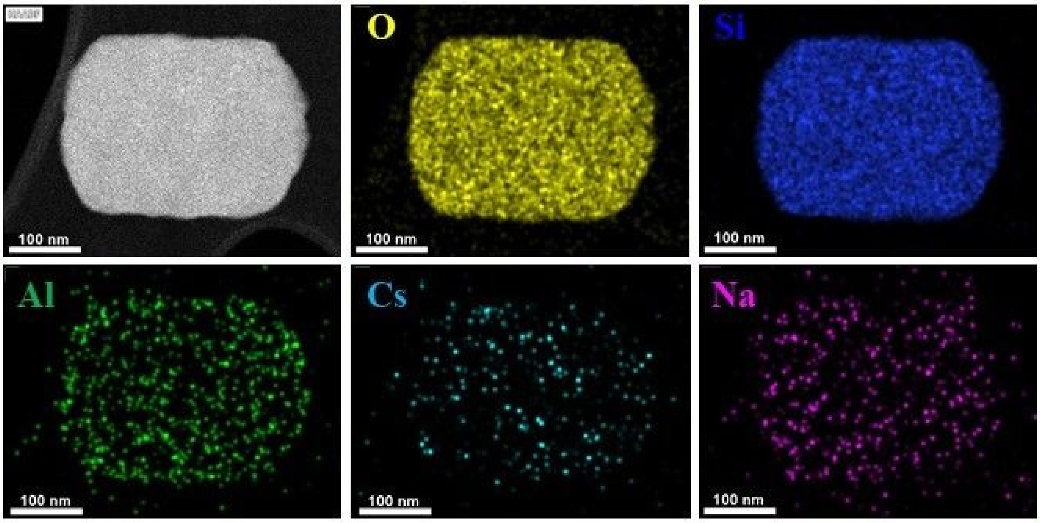

Figure S3: HAADF STEM and EDX-mapping of Cs-ZMS-5

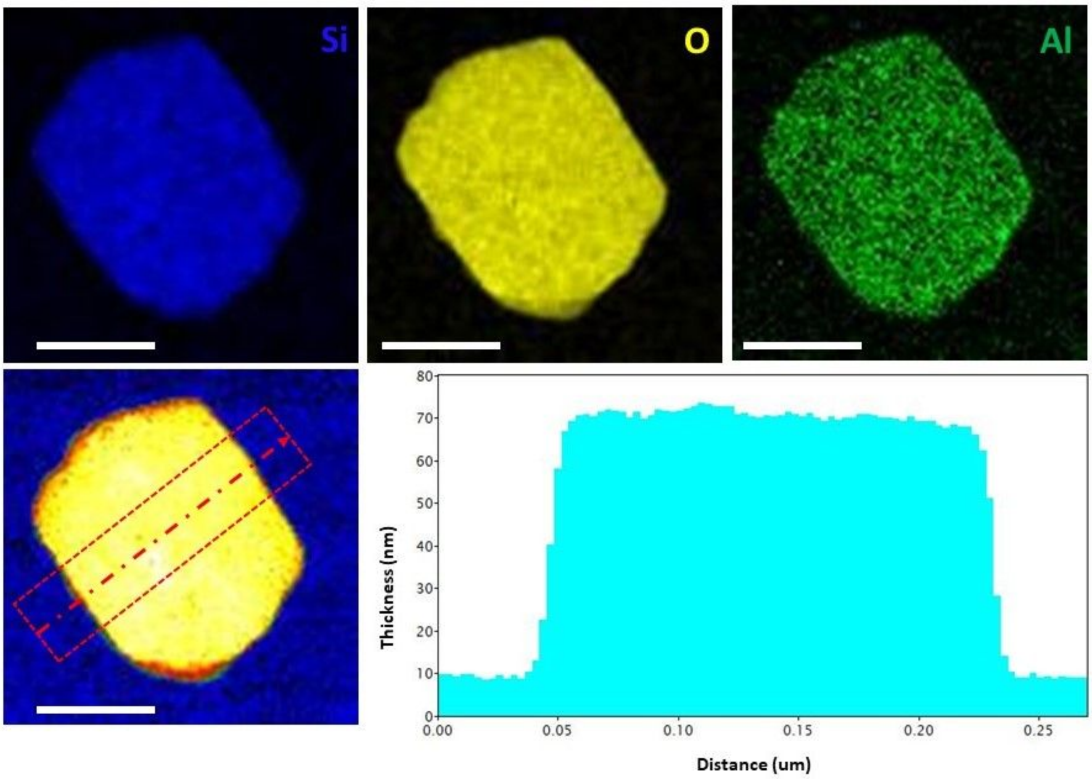

Figure S4: STEM-EELS elemental maps and thickness maps of ZMS-5. The scale bar is 100 nm.

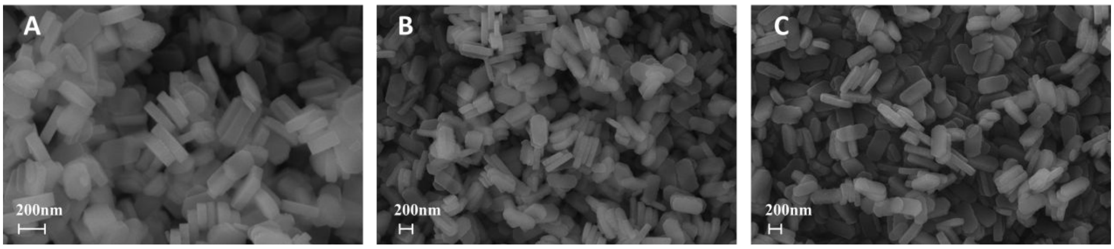

Figure S5: SEM images of A) Na-ZSM-5, B) Ba-ZSM-5, C) Cs-ZSM-5.

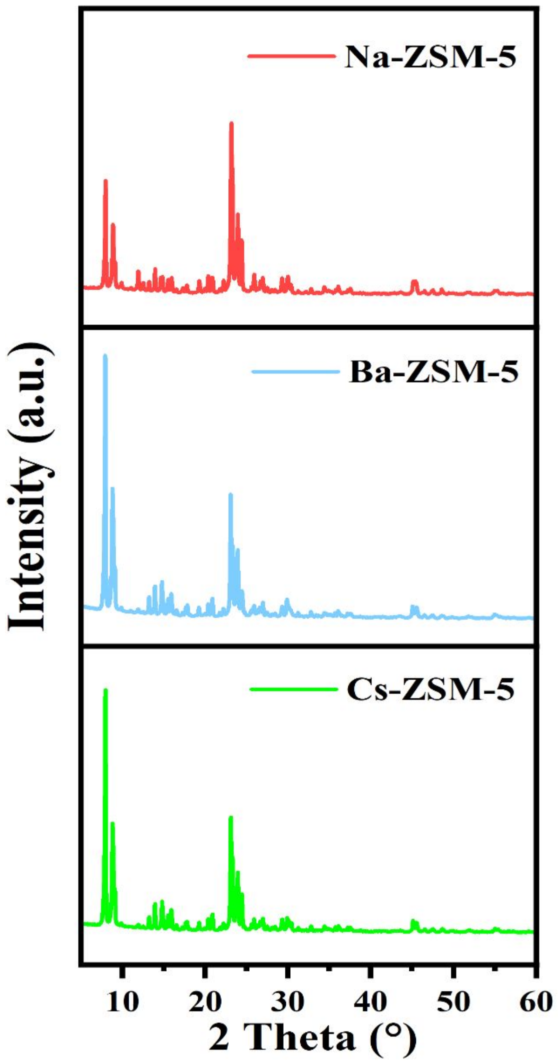

Figure S6: XRD patterns of Na-ZSM-5, Ba-ZSM-5, Cs-ZSM-5.

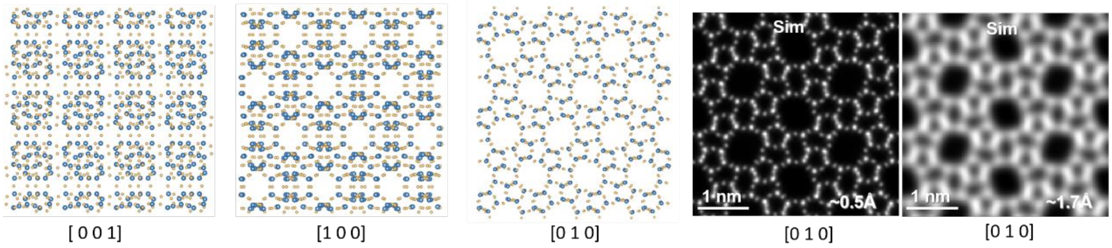

Figure S7: From left to right corresponding to structural model of the MFI-type zeolite framework from the [001], [100], [010] (this work) projection, simulated iDPC image with 0.5 Å, simulated iDPC image with 1.7 Å in [010] zone axis, respectively.

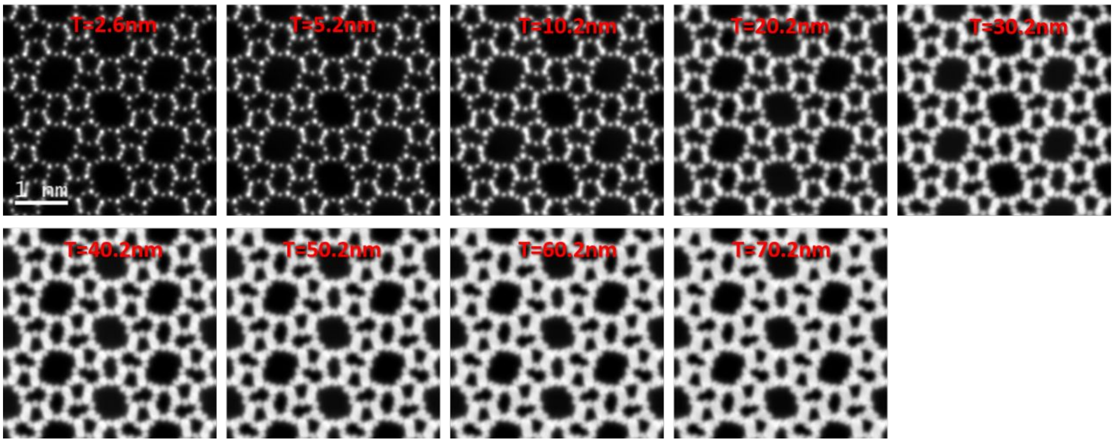

Figure S8: The simulated iDPC image of ZMS-5 over a thickness range of 2.6-70.2nm, where the spatial resolution is idealized at 0.5Å/50pm.

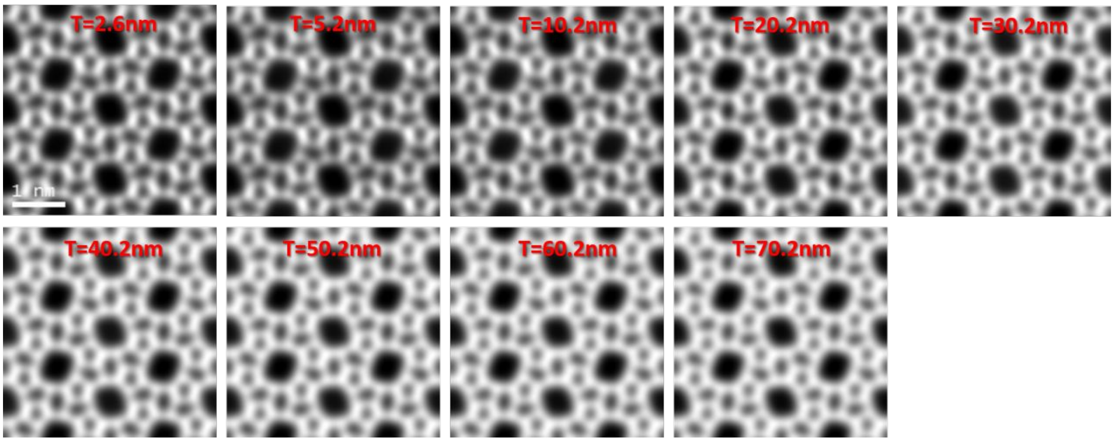

Figure S9: The simulated iDPC image of ZMS-5 over a thickness range of 2.6-70.2nm, where the spatial resolution followed up experimental at 1.7Å/170pm.

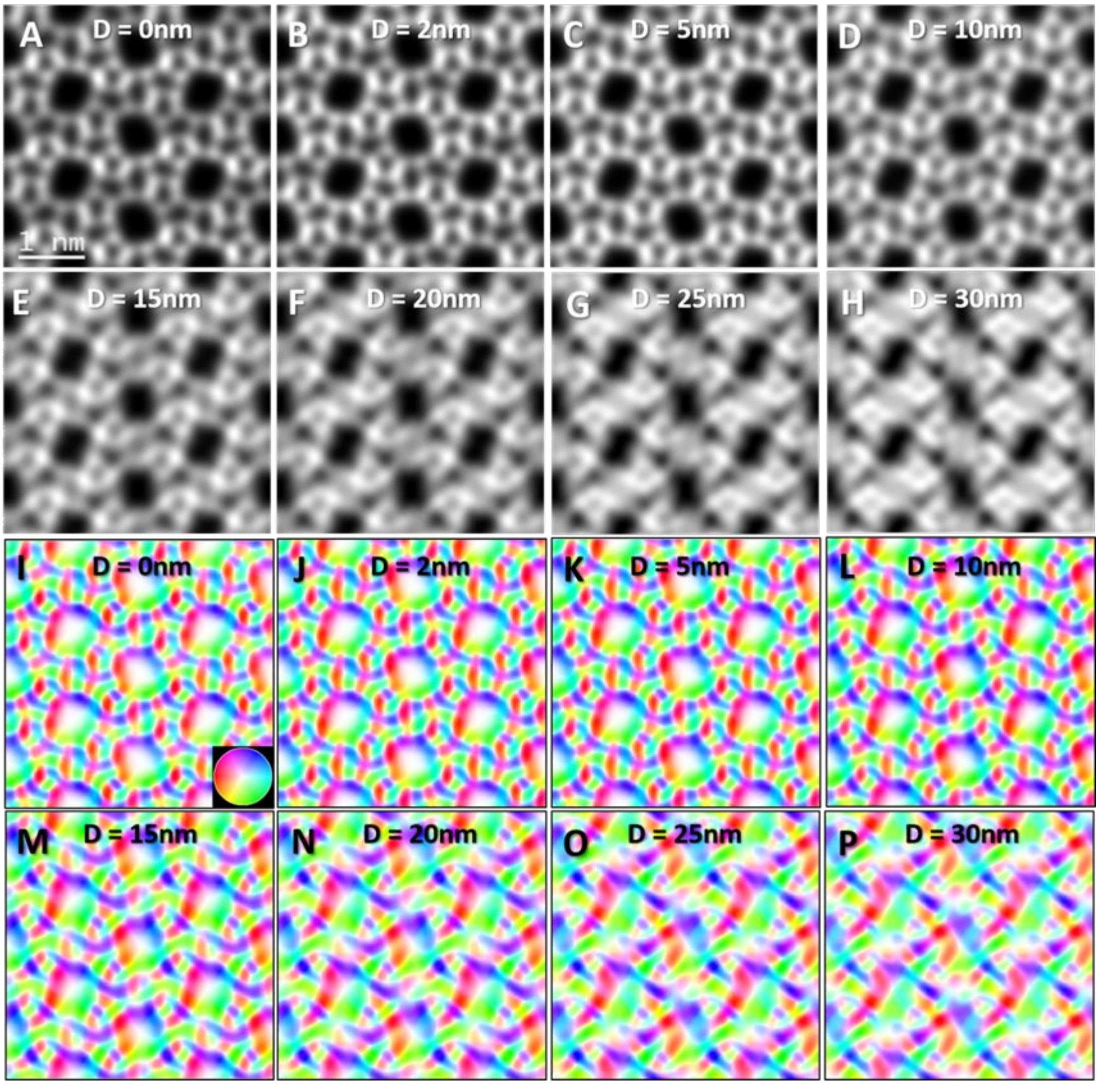

Figure S10: The simulated iDPC image (A-H) and DPC results (I-P) of ZMS-5 over defocus series ranging from 0 nm to 30 nm under experimental conditions, where the spatial resolution followed up experimental at 1.7Å/170pm.

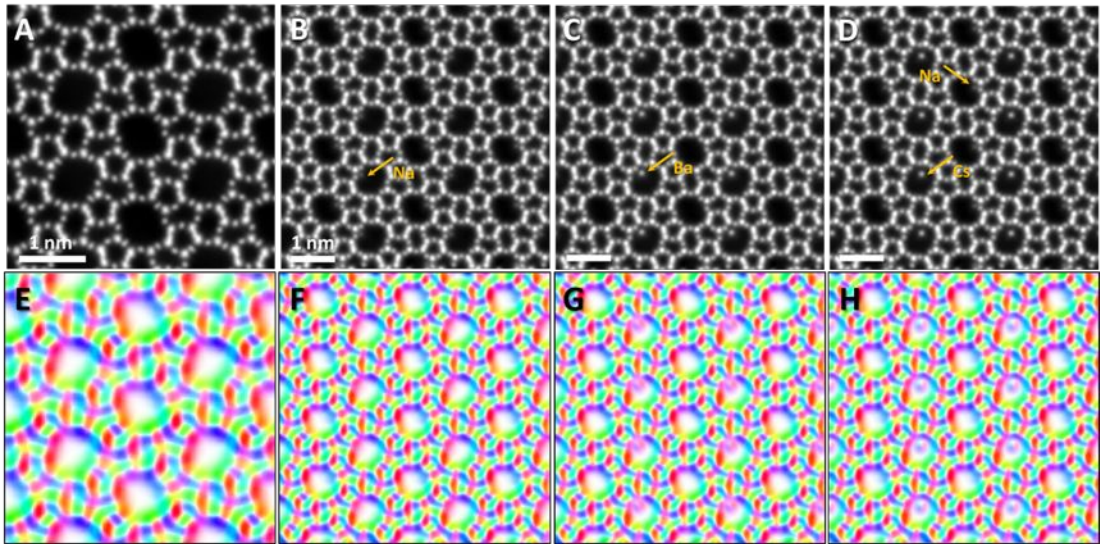

Figure S11: The simulated iDPC image (A-D) and DPC results (E-H) of ZMS-5, Na-ZMS-5, Ba-ZMS-5 and Cs-ZMS-5.

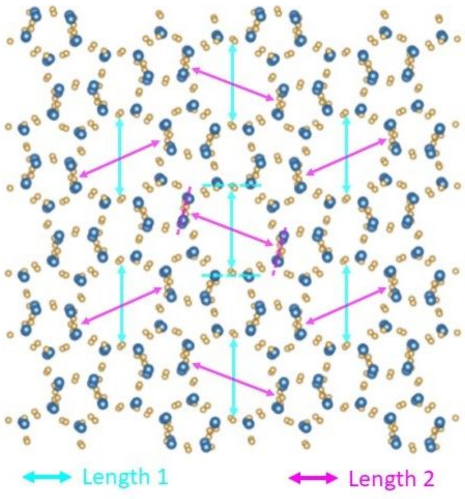

Figure S12: Definition of the length 1 and 2 in 10-MRs.

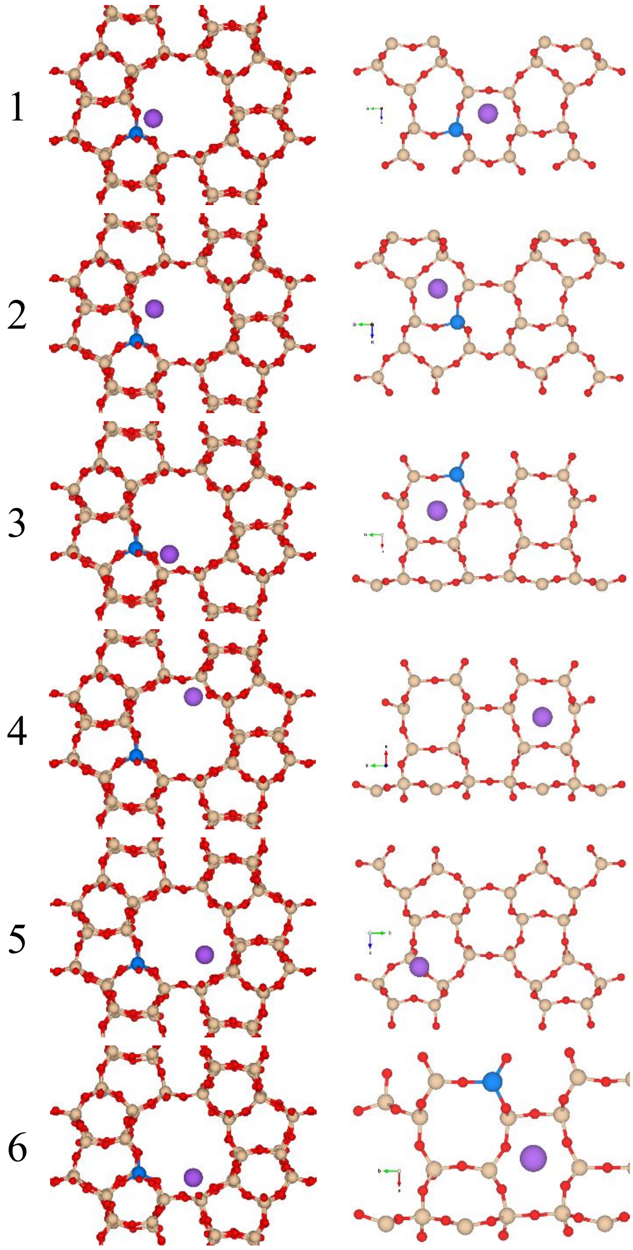

Figure S13: Structures of Na-ZSM-5 with six distinct cation positions investigated in this study. Red: O; light brown: Si; blue: Al; purple: Na.

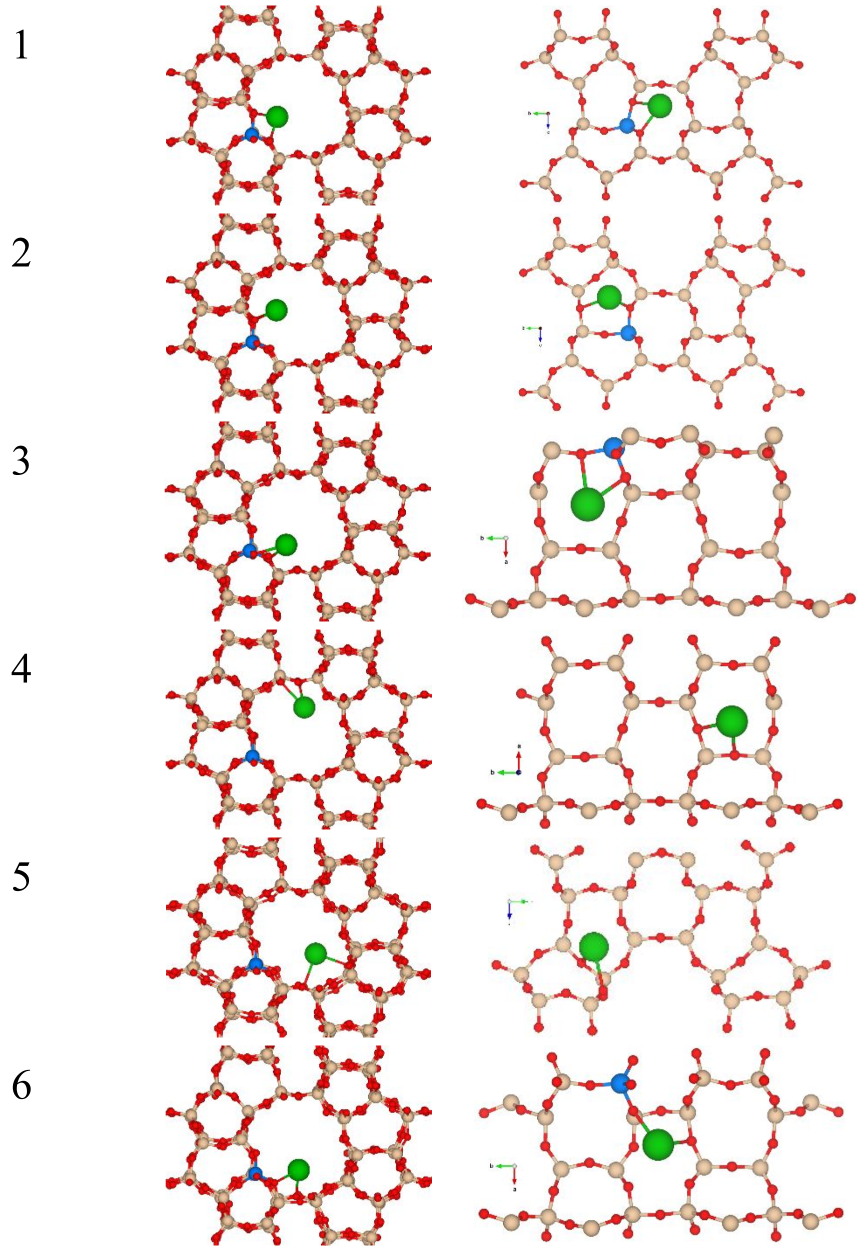

Figure S14: Structures of Ba-ZSM-5 with six distinct cation positions investigated in this study. Red: O; light brown: Si; blue: Al; green: Ba.

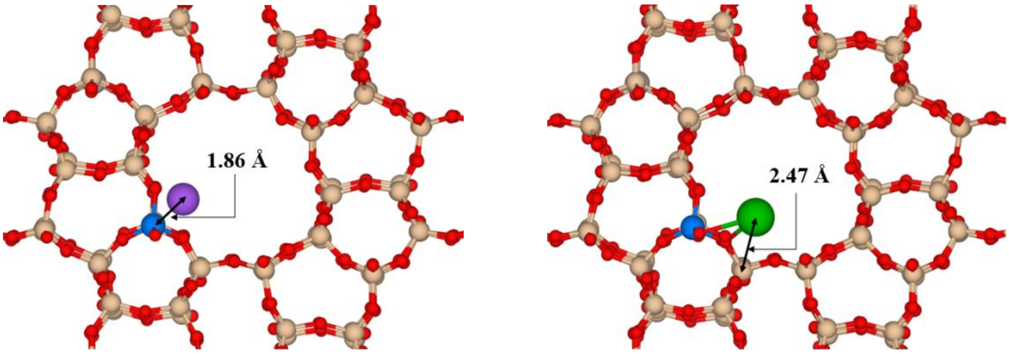

Figure S15: Distance between M^{n+} and the nearest T atom projected on [010] plane. A) Na-ZSM-5 B) Ba-ZSM-5. Red: O; light brown: Si; blue: Al; purple: Na; green: Ba.

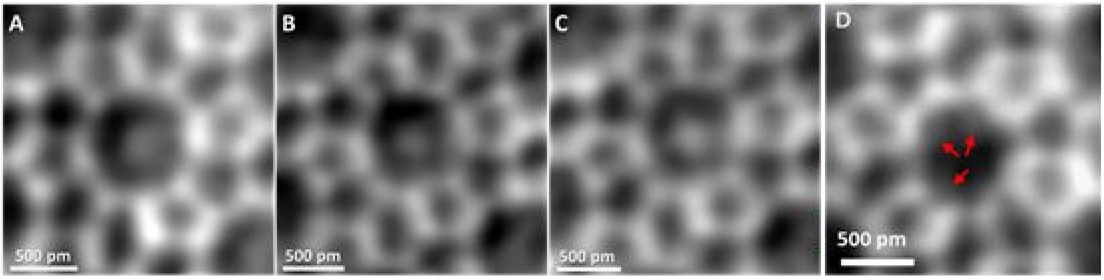

Figure S16: representative positions of Ba^{2+} in 10-MR frameworks (A-C), and typical positions of Na^{+} in 10-MR frameworks (D). Na^{+} was dotted with red arrow.

Discussion: As indicated, when Na^{+} is replaced by Ba^{2+}, the larger Ba^{2+} ions move farther from the edge of the 10-MR ring, suggested the distance between the cation (Mn^{+}) and the nearest T atom increased.

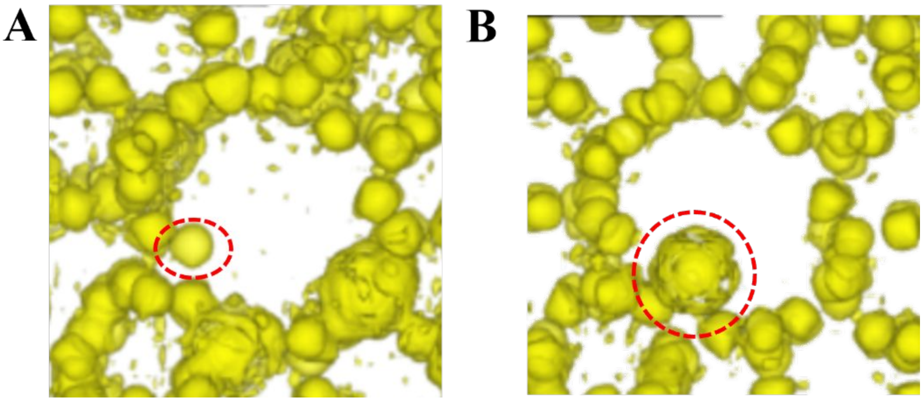

Figure S17: The difference electron density maps: (A) Na-ZSM-5, (B) Ba-ZSM-5.

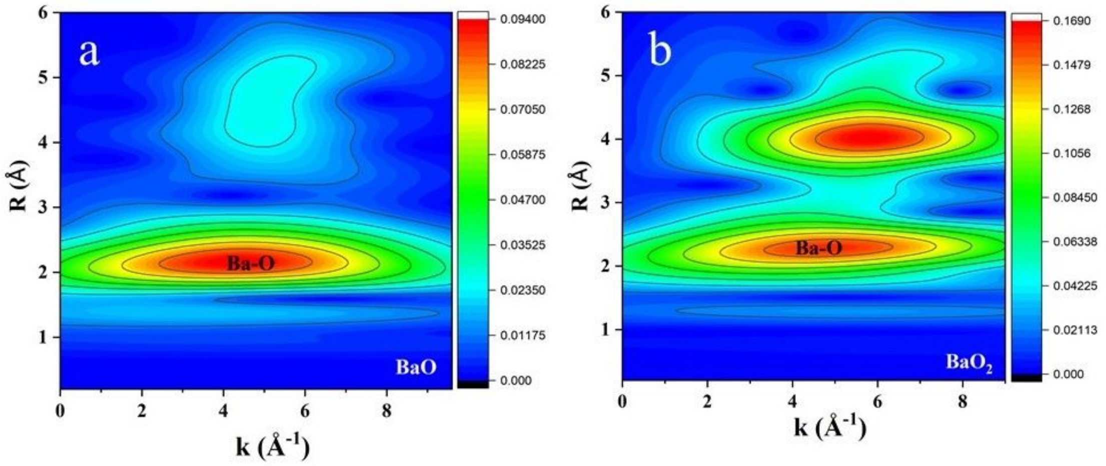

Figure S18: The wavelet transform analyses of the BaO (a), and BaO_2 (b) reference sample, respectively.

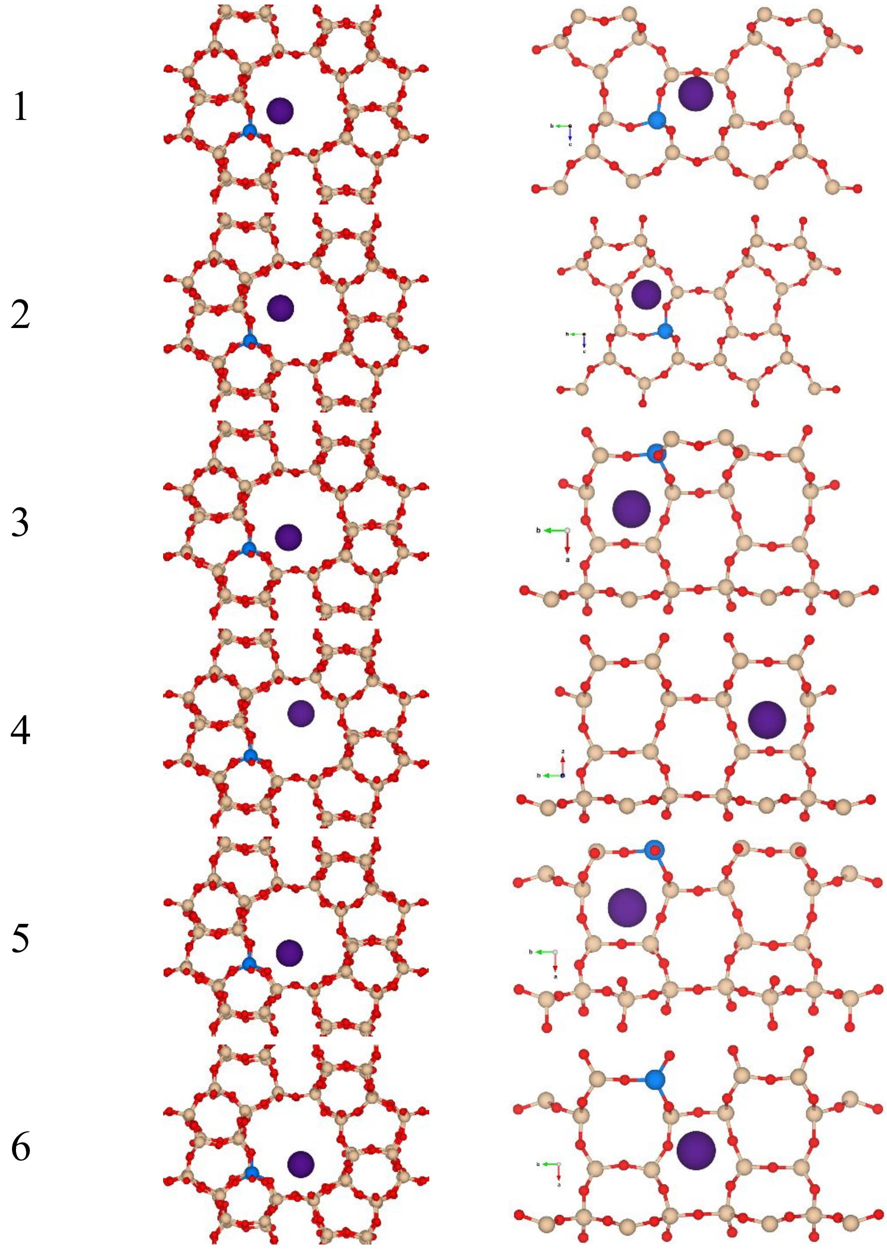

Figure S19: Structures of Cs-ZSM-5 with six distinct cation positions investigated in this study. Red: O; light brown: Si; blue: Al; dark purple: Cs.

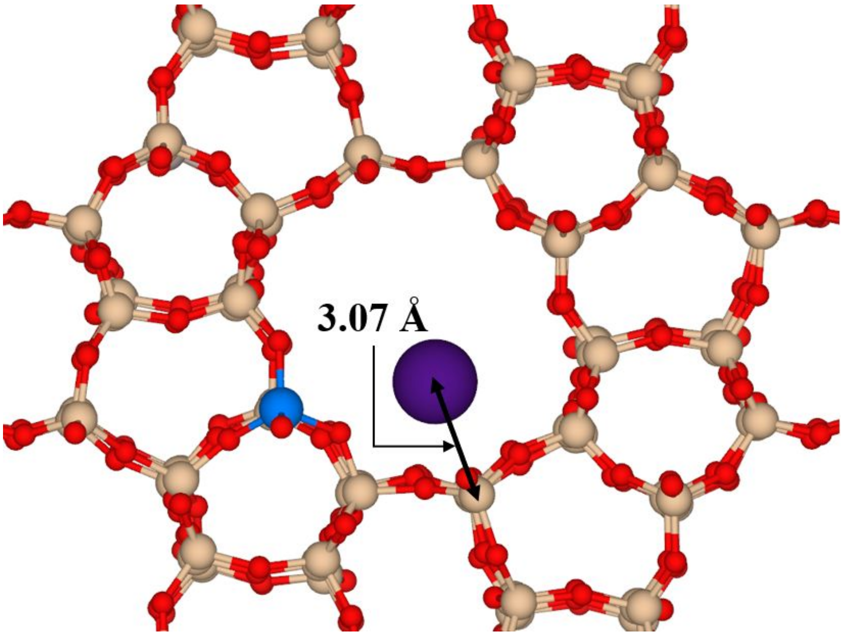

Figure S20: DFT calculation of the distance between the Cs^+ and the nearest Si atom in Cs^+-ZSM-5. Red: O; light brown: Si; blue: Al; dark purple: Cs.

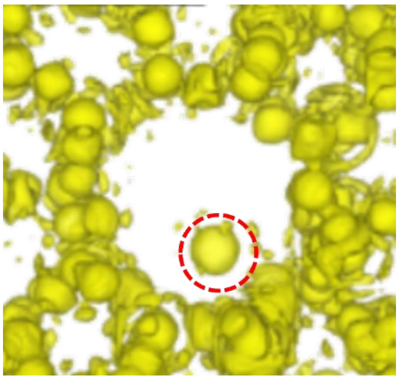

Figure S21: The difference electron density maps of Cs-ZSM-5.

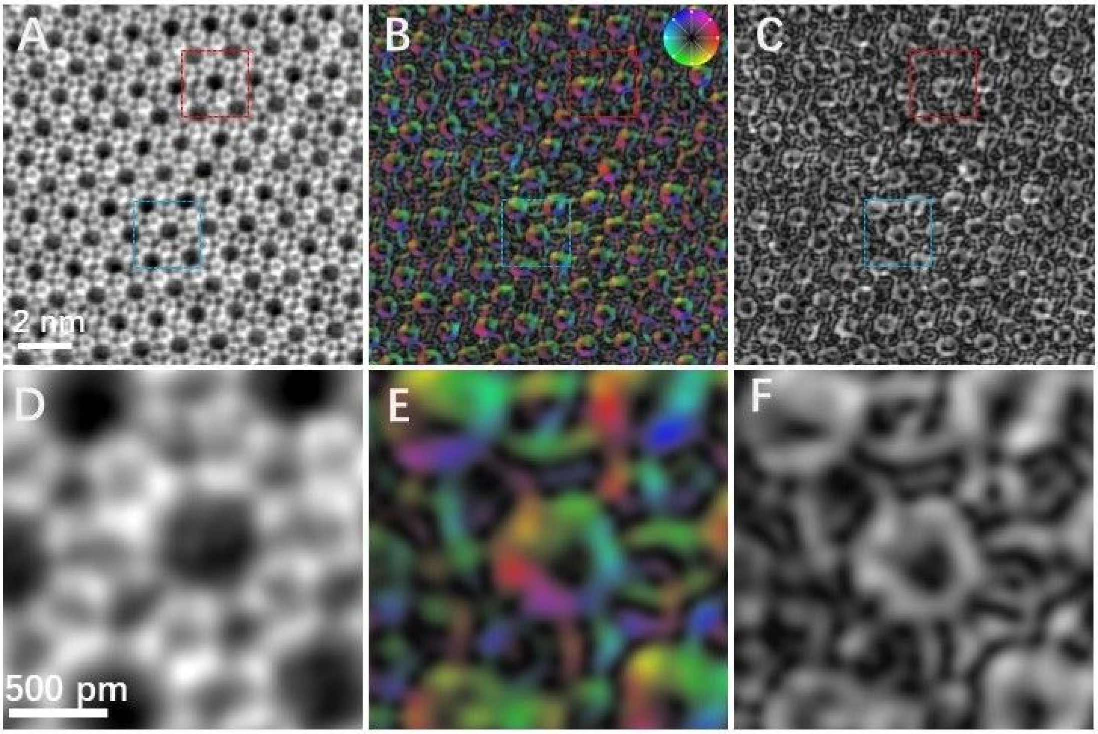

Figure S22: iDPC STEM image and DPC STEM map of Na-ZSM-5.

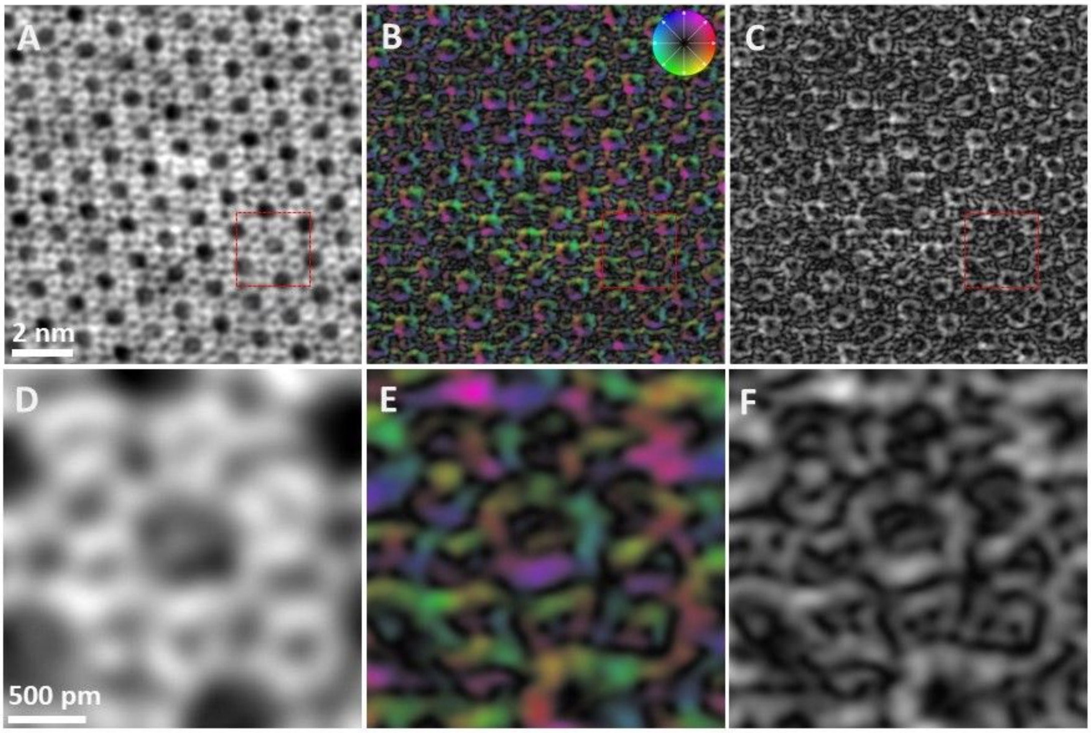

Figure S23: iDPC-STEM image and DPC STEM map of Cs-ZSM-5 with high-magnification.

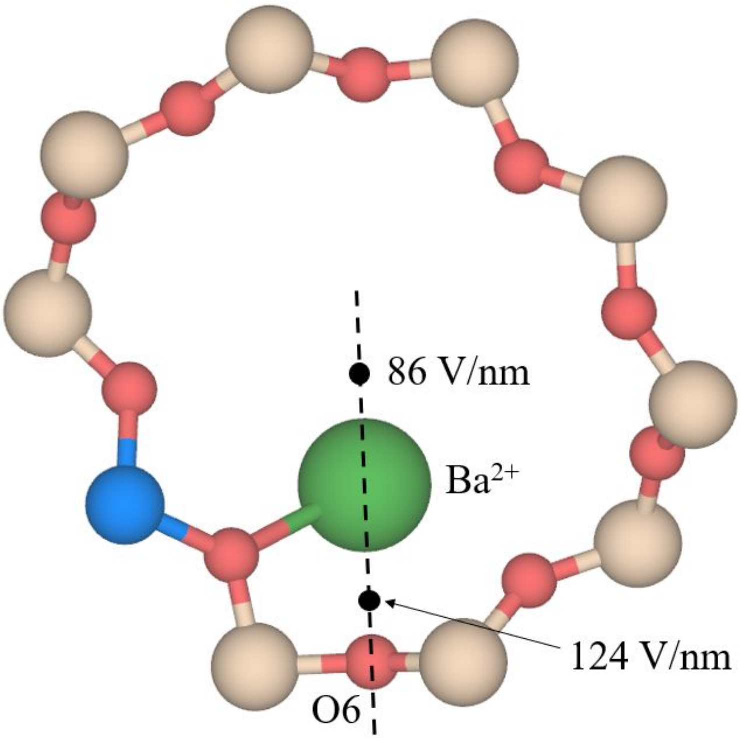

Figure S24: Total electric field strength projected on [010] plane at the midpoint of the M–O6 bond and at its symmetrically inverse position. Red: O; light brown: Si; blue: Al; green: Ba.

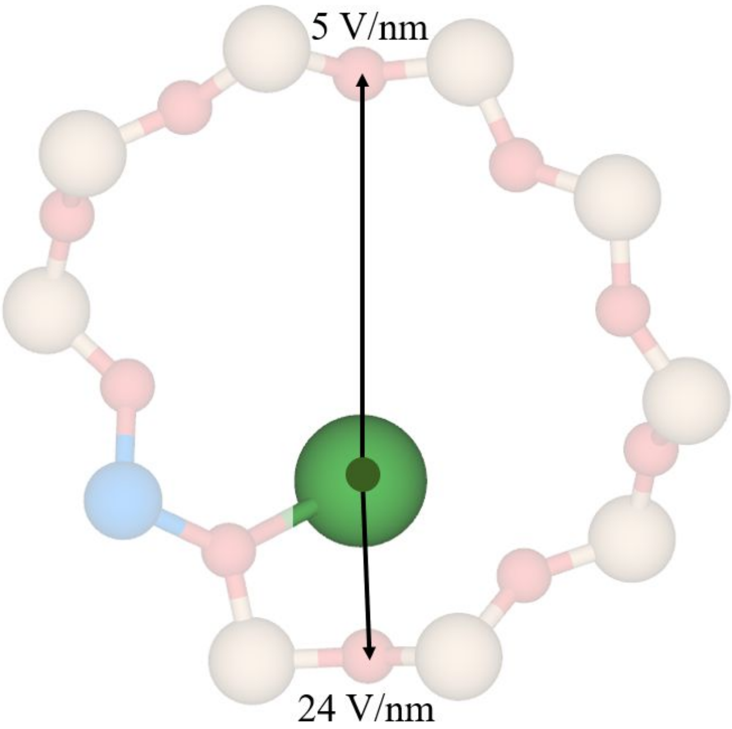

Figure S25: Strength of the electric field induced solely by Ba^{2+} at the adjacent O site and at the oxygen atom located on the opposite point of the 10MR. Red: O; light brown: Si; blue: Al; green: Ba.

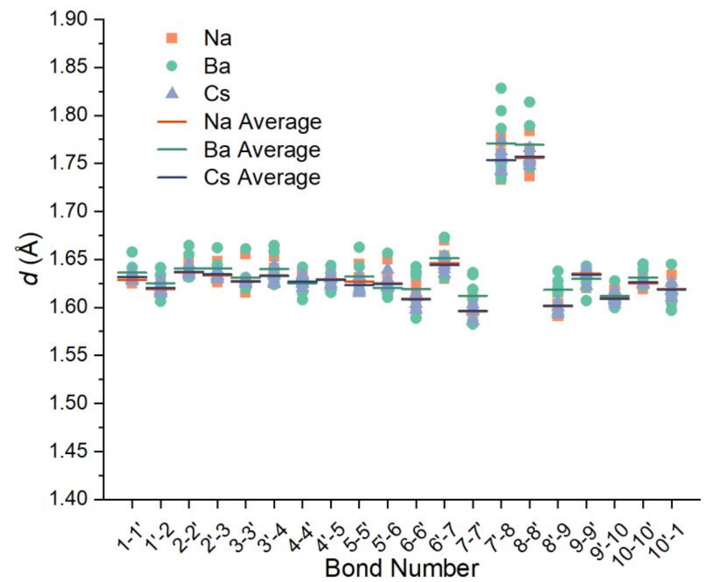

Figure S26: Distributions of Si-O distances between adjacent tetrahedral SiO_4 within 10-MR straight channel with different ions in DFT calcination.

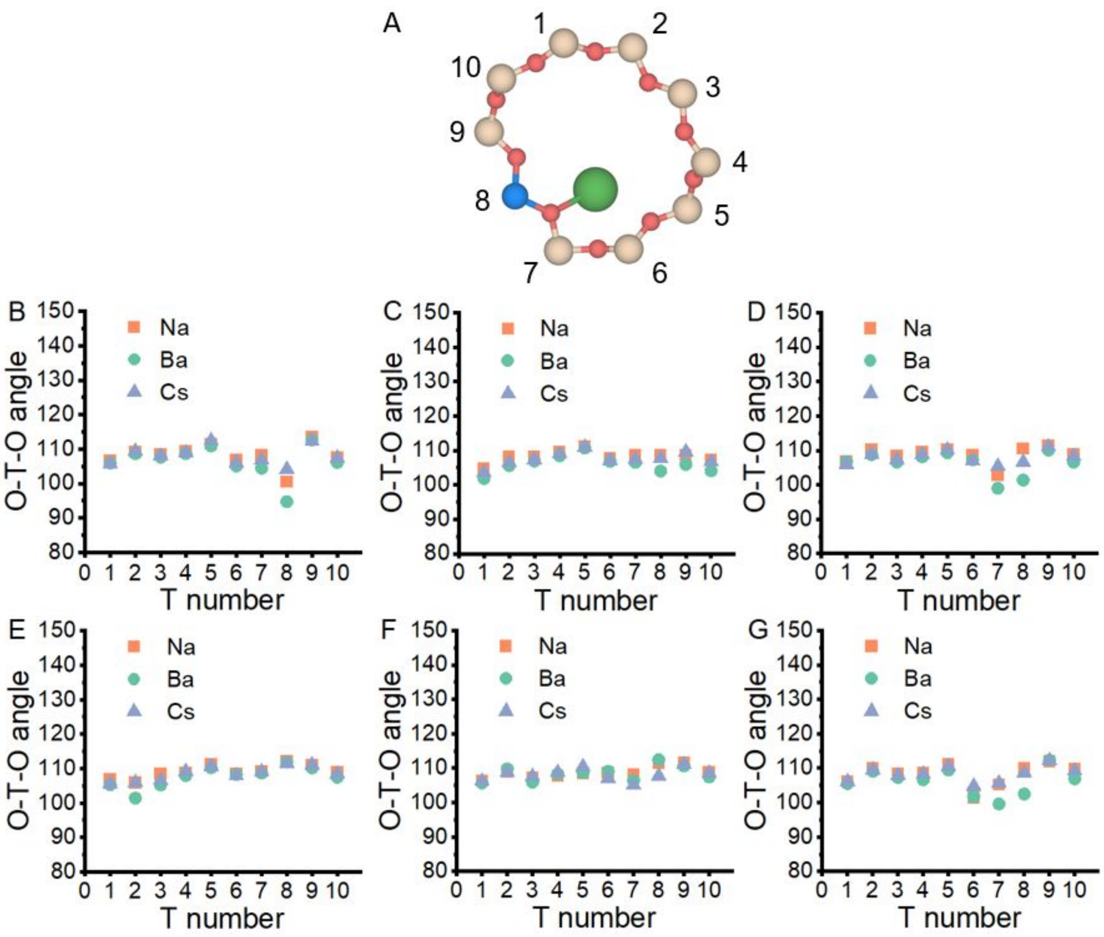

Figure S27: Distributions of T-O-T angles between adjacent tetrahedral TO_4 within 10-MR straight channel with different ions in DFT calculation. A) Numbering of O atoms. B) to G) T-O-T bonds on structure 1 to 6, respectively, as shown in Fig. S13, S14 and S19.

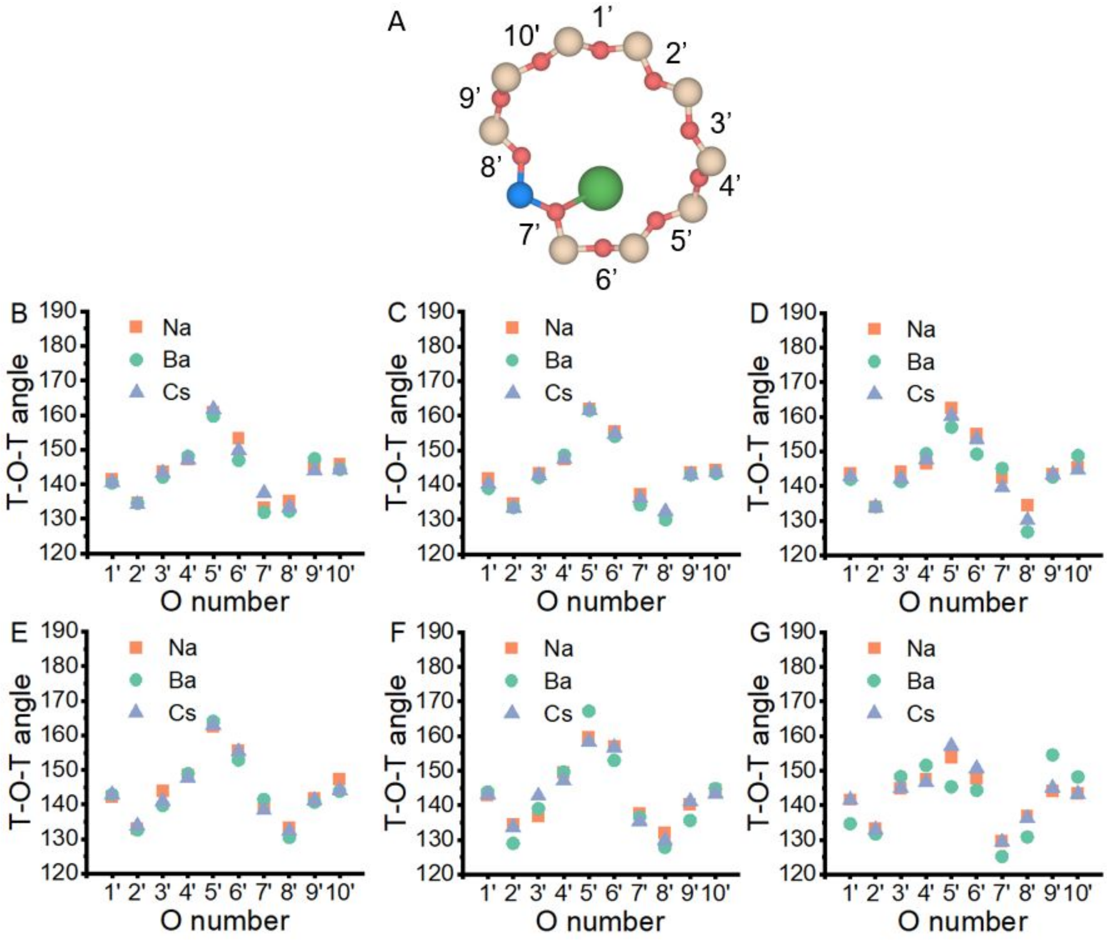

Figure S28: Distributions of O-T-O angles between adjacent tetrahedral TO_4 within 10-MR straight channel with different ions in DFT calcination. A) Numbering of O atoms. B) to G) O-T-O bonds on structure 1 to 6, respectively, as shown in Fig. S13, S14 and S19.

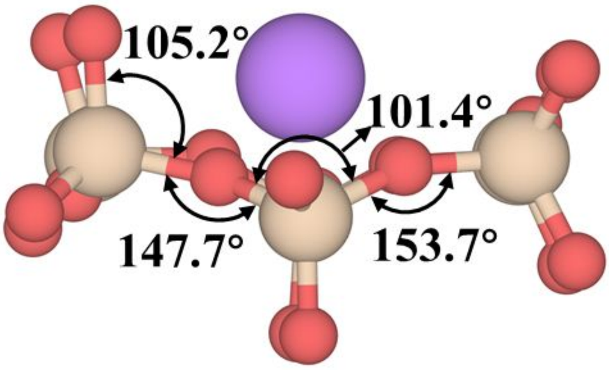

Figure S29: The bond angles in the detailed 10-MR pore structure of Na-ZSM-5.

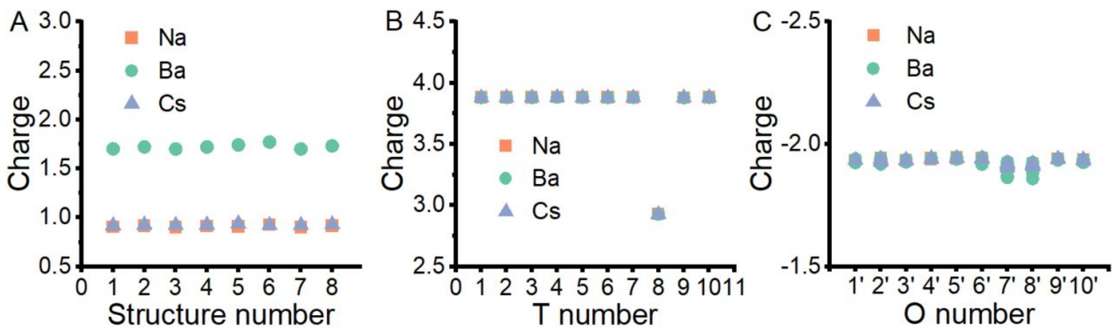

Figure S30: Bader charges on A) M^{n+} on all eight structures, B) on all T atoms at 10-MR at all 6 structures and C) on all O atoms at 10-MR at all 6 structures.

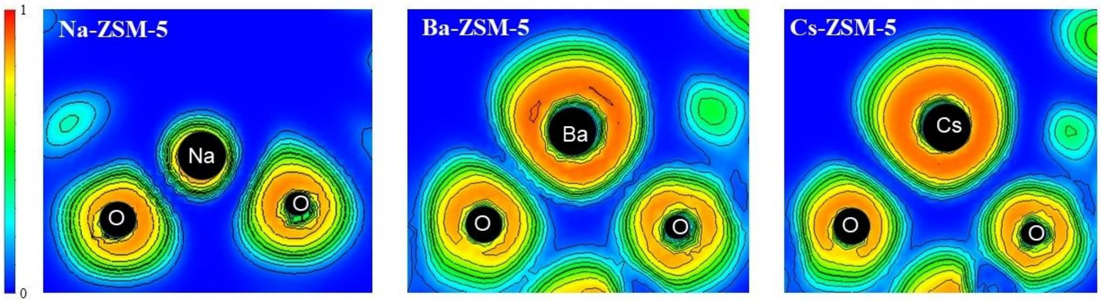

Figure S31: Electron localization function results of Na-ZSM-5, Ba-ZSM-5, Cs-ZSM-5, respectively.

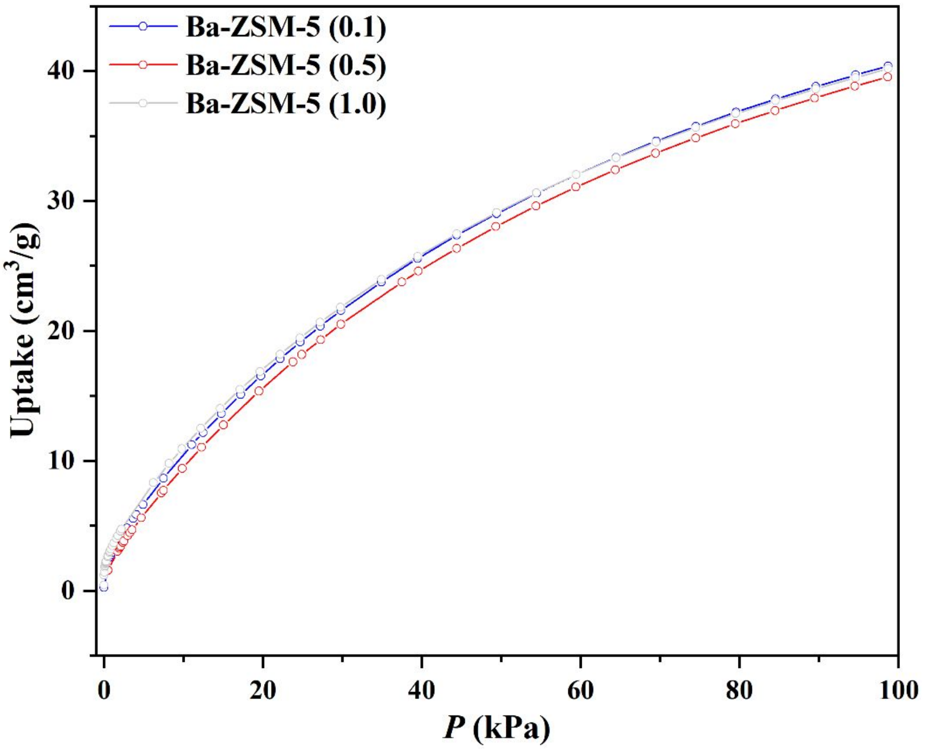

Figure S32: CO_2 adsorption isotherms of Ba-ZSM-5 at 298 K with different Ba^{2+} amounts.

Note: Based on XRF analysis, the exact content of Ba in the samples of Ba-ZSM-5 (0.1), Ba-ZSM-5 (0.5) and Ba-ZSM-5 (1.0) is 3.3%, 13.4% and 22%, respectively.

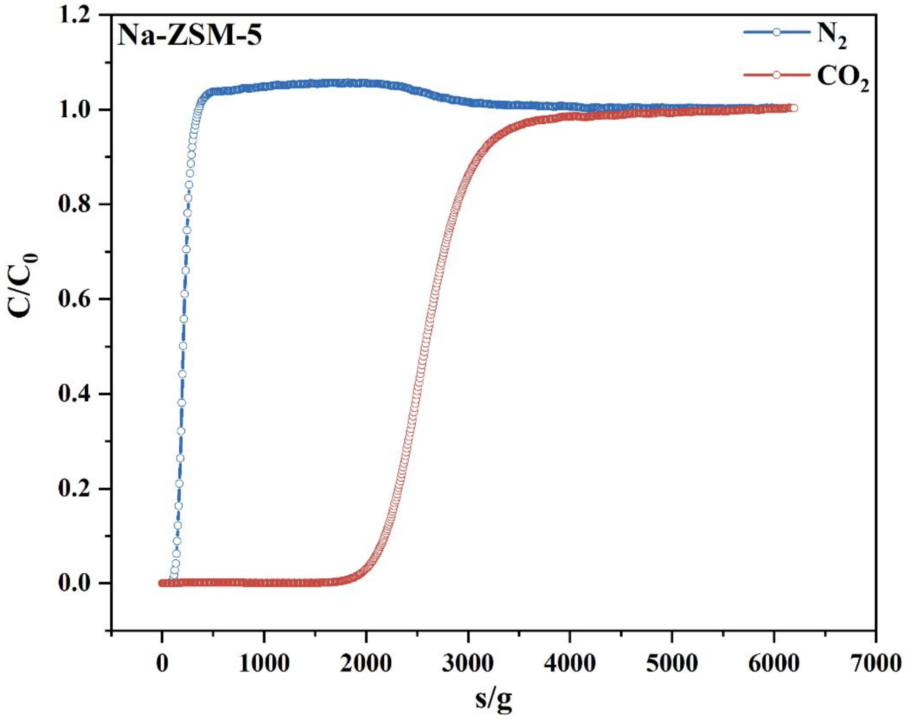

Figure S33: the breakthrough curve of CO_2/N_2 in Na-ZSM-5.

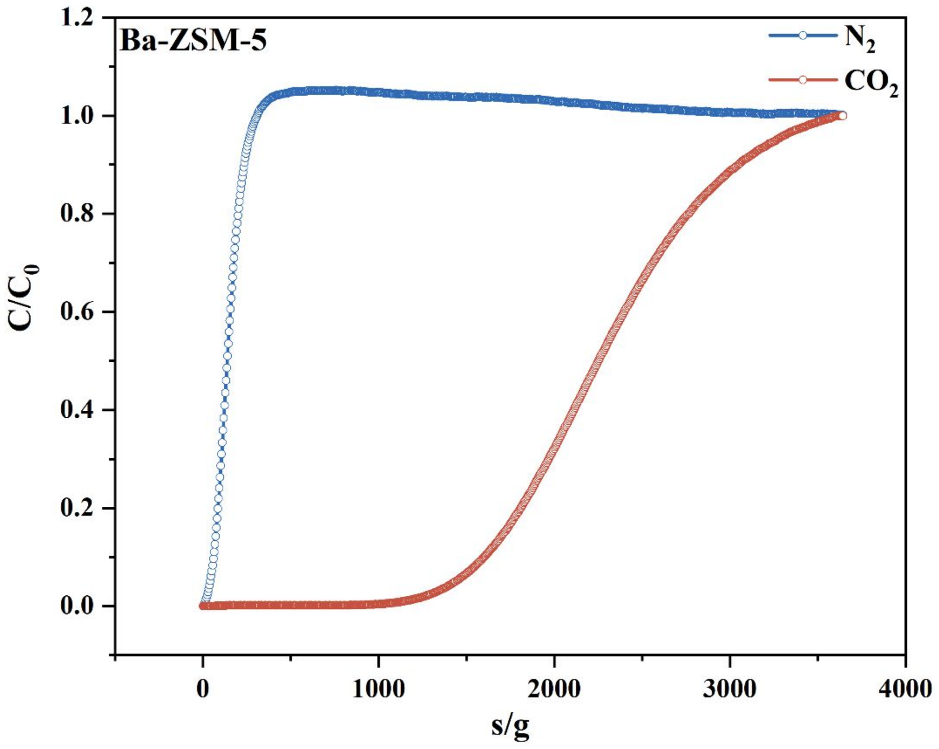

Figure S34: the breakthrough curve of CO_2/N_2 in Ba-ZSM-5.

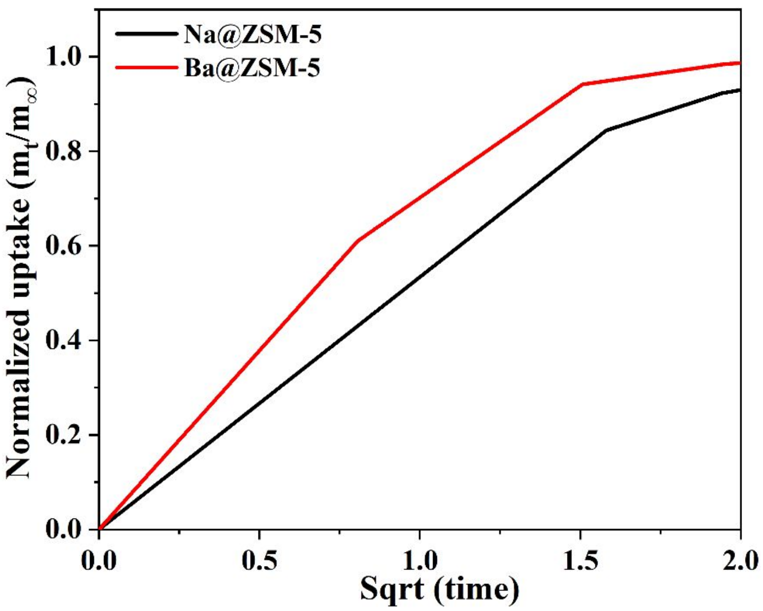

Figure S35: adsorption rate measurements on Na-ZSM-5 and Ba-ZSM-5.

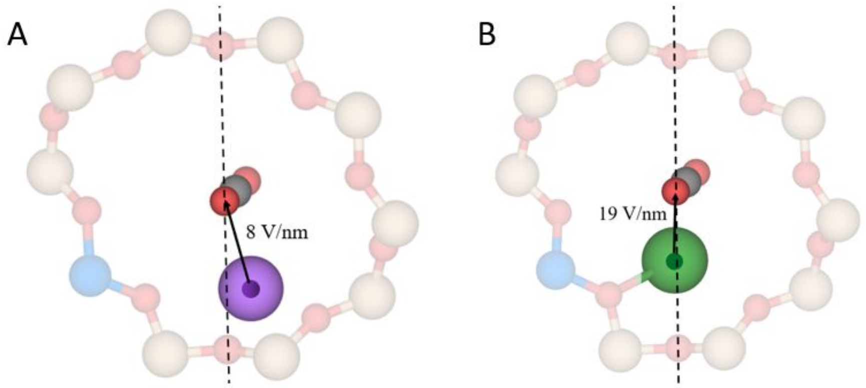

Figure S36: Electric field generated from A) Na^+ and B) Ba^{2+} at the center of the pore represented by the CO_2 molecule.

Table S1: Si/Al ratio in Na-, Ba-, Cs-, ZSM-5 zeolites

Sample	Al_2O_3 (wt%) [a]	SiO_2 (wt%) [a]	Si/Al_2 [b]
Na-ZSM-5	2.50	97.50	66
Ba-ZSM-5	1.62	62.18	65
Cs-ZSM-5	1.92	72.18	64

[a] Determined by mass fraction of Al and Si in zeolite samples via XRF characterizations.

[b] Mole ratio of SiO_2 to Al_2O_3.

Table S2: BET results of Na-, Ba-, Cs-, ZSM-5 zeolites

Sample	S_{BET} [a] (m^2 g^{-1})	S_{ext} [b] (m^2 g^{-1})	V_p [c] (cm^3 g^{-1})	$V_{meso+macro}$ [d] (cm^3 g^{-1})
Na-ZSM-5	417.3980	73.3371	0.144038	0.13241
Ba-ZSM-5	403.4968	62.2308	0.144882	0.112365
Cs-ZSM-5	402.7137	68.7008	0.137472	0.107553

[a] BET surface area.

[b] External surface area calculated using the *t*-plot method.

[c] Micropore volume calculated using the *t*-plot method.

[d] Mesopore and macropore volume calculated using the *t*-plot method.

Table S3: EXAFS fitting parameters at the **Ba** L3-edge for various samples

	Shell	*CN*[a]	*R*(Å)[b]	σ^2(Å^2)[c]	ΔE_0(eV)[d]	*R* factor
Ba-ZSM-5	Ba-O	2.0±0.17	2.78±0.01	0.0032	3.2	0.0076
	Ba-O	2.4±0.13	3.41±0.15	0.0068		
BaO	Ba-O	6*	2.77±0.03	0.0098	1.9	0.0153
BaO_2	Ba-O	4*	2.72±0.01	0.0026	7.9	0.0085
	Ba-O	4*	2.88±0.02	0.0070		

[a]*CN*, coordination number; [b]*R*, distance between absorber and backscatter atoms; [c]σ^2, Debye-Waller factor to account for both thermal and structural disorders; [d]ΔE_0, inner potential correction; *R* factor indicates the goodness of the fit.

Table S4: Description of the electric field for ZSM-5, Na-ZSM-5 and Ba-ZSM-5.

Testing Point*	Projected Electric Field Intensity (V/nm)			Angle Rotated(°)	
	ZSM-5	Na-ZSM-5	Ba-ZSM-5	ZSM-5 to Na-ZSM-5	Na-ZSM-5 to Ba-ZSM-5
M-T6		57	48		91.5
M-T7		107	50		111.5
M-O5		83	94		6.1
M-O6		134	124		28.4
M-O7		65	183		68.5
T1-O1	1051	989	943	2.9	1.2
O1-T2	1171	1172	1155	3.4	0.9
T2-O2	1146	1143	1142	2.3	0.4
O2-T3	1019	1052	1068	5.0	3.0
T3-O3	1155	1153	1152	1.8	1.7
O3-T4	1146	1148	1150	1.1	1.8
T4-O4	431	450	569	1.0	10.0
O4-T5	984	988	979	2.5	1.6
T5-O5	1142	1101	1081	1.0	2.8
O5-T6	992	993	993	4.9	6.8
T6-O6	1095	928	874	8.5	9.1
O6-T7	1105	1078	1081	10.2	2.4
T7-O7	1148	1224	1138	12.9	7.5
O7-T8	999	694	651	7.1	3.0
T8-O8	1147	813	767	2.6	6.0
O8-T9	1024	1091	1170	2.7	4.6
T9-O9	1063	1044	1027	5.6	8.8
O9-T10	645	623	635	11.1	17.1
T10-O10	1148	1139	1148	2.8	2.5
O10-T1	991	1020	1051	1.4	1.7

Table S5: The size list of ZSM-5 rings, gas and Ba^{2+}

Name	Size (nm)	Note(s)
10-MRs	~ 0.55	Pore size of 10-MRs from database[10]
6-MRs	0.26-0.28	Pore size of 6-MRs from database[10]
5-MRs	0.18-0.20	Pore size of 5-MRs from database[10]
Ba^{2+}	0.27	Diameter data of Ba^{2+} from database[11]
Rigid Ba^{2+}-10MR	0.28	Available space after Ba^{2+} occupancy in nominal rigid 10MR
		Calculation method: 0.55(rigid 10-MRs) - 0.27(Ba^{2+}) = 0.28nm
Flexible Ba^{2+}-10MR	0.33	Available space after Ba^{2+} occupancy in flexible 10MR
		Calculation method: 0.60(flexible 10-MRs) - 0.27(Ba^{2+}) = 0.33nm
CO_2	0.33	Kinetic diameter of CO_2 from database[12]

References

1. J. Barthel, Dr. Probe: A software for high-resolution STEM image simulation. *Ultramicroscopy*, **2018**, *193*, 1-11.
2. T. Malis, S. C. Cheng, R. F. Egerton, EELS log-ratio technique for specimen-thickness measurement in the TEM. *J. Electron Microsc. Tech.*, **1988**, *8*, 193-200.
3. J. P. Perdew, K. Burke, M. Ernzerhof, Generalized Gradient Approximation Made Simple. *Phys. Rev. Lett.*, **1996**, *77*, 3865.
4. S. Grimme, Supramolecular Binding Thermodynamics by Dispersion-Corrected Density Functional Theory. *Chem. Eur. J.*, **2012**, *18*, 9955-9964.
5. M. Maestri, E. Iglesia, First-principles theoretical assessment of catalysis by confinement: NO–O2 reactions within voids of molecular dimensions in siliceous crystalline frameworks. *Phys. Chem. Chem. Phys.*, **2018**, *20*, 15725-15735.
6. P. Giannozzi, O. Baseggio, P. Bonfà, D. Brunato, R.Car, I. Carnimeo, C. Cavazzoni, S. de Gironcoli, P. Delugas, F. Ferrari Ruffino, A. Ferretti, N. Marzari, I. Timrov, A. Urru, S.Baroni, Quantum ESPRESSO toward the exascale. *J. Chem. Phys.*, **2020**, *152*, 154105.
7. X. Wang, Z. Liu, J. Wang, T. Lu, W. Xiong, X. Yan, M. Zhao, M. Orozco-Ic, *Chem. Eur. J.*, **2023**, *29*, e202300348.
8. W. Tang, E. Sanville, G. Henkelman, A grid-based Bader analysis algorithm without lattice bias. *J. Phys.: Condens. Matter.*, **2009**, *21*, 084204.
9. F. Neese, The ORCA program system. *WIREs Comput. Mol. Sci.*, **2012**, *2*, 73-78.
10. https://www.iza-structure.org/databases/
11. https://www.webelements.com/barium/atom_sizes.html
12. https://www.chemxin.com/common-molecular-diameters.html
